# Evaluating the Accuracy of Bonded Block Models for Prediction of Rockmass Analog Mechanical Behavior

**DOI:** 10.3390/ma17010088

**Published:** 2023-12-23

**Authors:** Isabella West, Gabriel Walton, Sankhaneel Sinha

**Affiliations:** 1 Department of Geology and Geological Engineering, Colorado School of Mines, Golden, CO 80401, USA; igwest@mines.edu (I.W.); sankhaneelsinha@mines.edu (S.S.); 2 WSP USA Inc., Lakewood, CO 80226, USA; 3 Equilibrium Mining, Kolkata 700042, India

**Keywords:** rockmass behavior, numerical modeling, mechanical properties, triaxial testing, bonded block models, UDEC Voronoi, artificially jointed specimens, forward prediction, prediction model, synthetic rockmass model

## Abstract

Large-scale rock formations, referred to as “rockmasses”, consist of intact rock separated by pre-existing discontinuities (i.e., joints). The mechanical behavior of rockmasses is difficult to directly test in the laboratory due to the required specimen scale. Instead, Synthetic Rockmass Modeling (SRM) is often used to simulate field-scale rockmass behavior. SRM requires a calibrated discrete element model (DEM) of intact rock combined with a Discrete Fracture Network (DFN). While the SRM concept has been informally determined to provide reasonable results based on practitioner experience, detailed and peer-reviewed validation is lacking. The goal of this study was to evaluate the predictive capabilities of the SRM method. Previously available data on intact and rockmass analog laboratory specimens of Blanco Mera granite containing DFNs with two joint sets were used as a basis for the SRM created in this study. Specifically, the intact DEM was a Bonded Block Model (BBM), generated to match the grain structure and composition of Blanco Mera granite and the model’s input parameters were calibrated so that the behavior of the BBM matched that of the intact laboratory specimens. The predictive capabilities of the model were evaluated by recreating the DFN from the jointed laboratory specimens within the intact BBM and comparing the behavior of the jointed models back to the jointed laboratory specimens, which has not been previously studied in the literature. The BBM was found capable of approximately predicting the behavior of rockmass analog specimens containing a pre-existing DFN without further calibration, which shows potential for the use of SRM in both industry and academia. Specifically, the BBM predicted the strength, dilatancy, and microfracturing behavior of the jointed laboratory specimens.

## 1. Introduction

Excavation of rock is necessary for the construction of many engineering structures like deep foundations, road cuts, and surface and underground mines. At scales relevant to these engineering structures, rock is not a homogeneous, intact material; it contains pre-existing structural discontinuities like joints, shear zones, etc. These structural discontinuities reduce the strength of the material compared to purely intact rock and also change other aspects of its mechanical behavior [1]. Rockmasses can be characterized mechanically by attributes such as strength, stiffness, and brittleness. Such material attributes can be estimated through laboratory testing on representative rockmass specimens.

However, since field-scale rock formations (i.e., rockmasses) are composed of sections of intact rock separated by a network of pre-existing discontinuities (e.g., joints, faults), direct experimental research on rockmass mechanical behavior is limited due to the large sampling scale necessary to adequately represent most rockmasses. Although large-scale laboratory apparatus exist, they typically allow for specimens up to only 1 m in diameter [2,3,4,5]. Additionally, such large-scale testing is expensive and difficult to perform.

Due to these scale limitations, two alterative techniques for researching rockmass behavior have been used in the literature: (1) testing of laboratory-scale rockmass analog specimens (pre-existing joints sawed/cured into small-scale intact rock/material core), where the influence of pre-existing joints on mechanical behavior can be directly evaluated in the laboratory [6,7,8,9,10,11] and (2) Synthetic Rockmass Modeling (SRM), which uses numerical models to simulate rockmasses at the field-scale [12,13,14].

SRM avoids the practical limitations associated with laboratory testing as rockmasses with any joint geometry and properties can be easily simulated using numerical models. The concept of SRM involves the use of a calibrated discrete element model of intact rock, typically calibrated based on laboratory data (at the decimeter scale) [15]. A discrete fracture network (DFN) of pre-existing joints is incorporated within the calibrated intact material and used to predict the behavior of the rockmass at large scales and/or with the presence of pre-existing joint sets [16]. SRM is based on the premise that if a model can be calibrated so that the micromechanical damage mechanisms (i.e., inelastic yield of blocks and the formation of cracks along block boundaries) occurring at a small-scale in real rock, in addition to matching emergent material attributes (i.e., strength, stiffness, dilatancy), then the model will act in a realistic manner under other conditions. Although numerous SRMs have been developed in the literature that have applied the SRM approach (as well as rock mechanics modeling studies more generally), they have either focused on back-analysis/calibration [14,17,18,19] or practical forward modeling in field-scale case studies [12,13,20,21,22]. Such studies have performed limited and/or qualitative comparison to field data thus do not provide a rigorous evaluation of model predictive performance. Typically, the DFNs used in literature are complex, modeled after in situ rockmasses (and often after case studies) [20,21,23]. However, direct and controlled validation of these large-scale and complex SRMs is not possible, as such rockmasses cannot be tested in the laboratory. Accordingly, the goal of this study is to evaluate the predictive capabilities of SRM using laboratory-scale rockmass analog specimens. Such an evaluation has not, to the best of our knowledge, been documented in the literature.

The laboratory data from [24], a previously published study using rockmass analog laboratory specimens of Blanco Mera granite containing smooth, saw-cut joints, have been used as the basis for the SRM developed in this study. Bonded Block Modeling (BBM) was the modeling method chosen for SRM development, as it has been demonstrated to be an effective approach for modeling laboratory triaxial compression tests on intact rock [25,26,27,28,29,30].

Numerical models—both BBMs and otherwise—have been previously created and calibrated to the Blanco Mera granite laboratory data from Ref. [24] [17,18,19,31,32,33]. However, each of these studies calibrated their models to both intact and jointed specimen data simultaneously, which does not ensure that the model is predictive under conditions different from which the model was calibrated for [34] (see Section 3.5 for further discussion on this topic). This method of using a single data set to both calibrate and evaluate the model performance is common practice in other SRM scenarios as well, both at the laboratory-scale [35,36] and the field-scale [14,17,18,19]. In contrast, the goal of the current study was to create a BBM of intact Blanco Mera granite calibrated to intact laboratory data of [24] and evaluate its predictive capabilities to match laboratory behavior of the jointed rockmass analog specimens.

In this study, a BBM was generated to match the intact rock grain structure of Blanco Mera granite and the model input parameters for two different models (with elastic or inelastic blocks) were calibrated so that the behavior of the intact model matched that of the intact laboratory data. Next, pre-existing smooth joints were added to the BBM in the same locations and orientations as the joints in the jointed laboratory specimens from [24] and no further calibration was performed. The mechanical behavior of the jointed models was then compared to the behavior of the jointed laboratory specimens to evaluate the ability of the two different models (elastic and inelastic blocks) to predict rockmass analog mechanical behavior outside the conditions for which the initial model was calibrated.

The remainder of this paper is organized into six main sections: (1) background information related to the Bonded Block Modeling (BBM) method applied in this study; (2) relevant information on previous laboratory testing and numerical modeling of Blanco Mera granite; (3) an overview of the methods performed to generate and calibrate the BBM; (4) the results of the BBM calibration; (5) the evaluation of the predictive capabilities of the BBM for jointed specimen cases; and (6) discussion of the results and their future implications. Ultimately, the comparison between model predictions and laboratory data for the jointed specimen cases presents a direct evaluation of the predictive capabilities of the type of BBMs created in this study, which has not been previously documented in the literature.

We note that the development of the model geometry used in this study and details of the calibration of the intact Blanco Mera granite BBM with elastic blocks presented in this paper were previously presented by [37]. Critical aspects of this previous work are summarized in this paper. The calibrated intact BBM with inelastic zones and the jointed model results are fully unique to this study.

## 2. Background on Bonded Block Modeling (BBM)

A common discrete element modeling (DEM) approach used to represent intact rock in SRMs is Bonded Block Modeling (BBM) [29,38,39,40]. BBMs have been demonstrated to be effective for modeling laboratory triaxial compression tests on intact rock [25,26,27,28]. Due to its ability to be used in both field-scale and laboratory-scale applications, BBM was the chosen method used to develop the models in this study.

In BBMs, material is broken into a set of distinct, polygonal elements that are initially bonded to and interact with each other via contacts. BBMs are effective at modeling the mechanical behavior of intact rock because the polygonal blocks can be generated with specified shapes and sizes that can reasonably approximate the grain structure of low-porosity rocks [41,42]. At the laboratory-scale, blocks can be assigned as certain minerals to match the composition of the real rock and therefore the interaction of the blocks with adjacent ones is analogous to the interaction between mineral grains within real rock [25]. Contacts of blocks within BBMs can break, simulating the loss of strength that occurs at grain-to-grain contacts in rock as damage accumulates. Therefore, BBMs simulate material failure through the formation and propagation of grain-scale fractures along the block boundaries [28].

The blocks in a BBM can be modeled as elastic or inelastic. Elastic blocks have been most commonly used in the literature [25,26,27,30,42,43,44,45], but BBMs with inelastic blocks have been found to more accurately replicate the behavior of intact rock, particularly in the post-peak regime and under higher confinements [28,46]. This is because the use of inelastic blocks allows the model to replicate the increased intragranular deformation and damage that occurs under these conditions. As both block types can be reasonably used in different scenarios (and the simpler elastic blocks are preferable in cases where they are applicable), two BBMs were created for this study: one using elastic block and the other using inelastic blocks.

As discussed previously, BBMs simulate material failure through the formation and propagation of grain-scale fractures along the block boundaries (i.e., “contacts”). Both the elastic and inelastic block BBMs in this study use an elastoplastic constitutive model to define the deformation of the contacts (i.e., the boundary between two adjacent blocks). The strength of the contacts is defined using the Mohr–Coulomb failure criterion and both peak and residual strength properties are assigned to the contacts, where the strength of the contacts instantaneously decrease to residual levels after their peak strength has been reached (the “Coulomb Slip Model” per [47]). Please refer to [47] for a complete explanation of the mechanisms involved in the Coulomb Slip Model and other constitutive models available in UDEC.

Deformation also occurs within blocks in a BBM. The difference between elastic and inelastic blocks is the constitutive models used to define the deformation of the blocks, which are governed by different sets of input parameters. Elastic blocks use an elastic, isotropic model where the blocks can deform elastically but have infinite strength. This constitutive model requires only density and elastic moduli as inputs. On the other hand, inelastic blocks use an elastoplastic constitutive model, which requires density, elastic moduli, strength, and post-yield dilation input parameters. Specifically, the Mohr–Coulomb criterion was used to define the peak and residual strengths of the inelastic blocks (the “Strain-Softening Mohr–Coulomb Model” per [47]). Per this elastoplastic constitutive model, inelastic blocks will first deform elastically, defined by their elastic moduli inputs. With further loading, blocks begin to experience inelastic (i.e., permanent) deformation once the stress within the block becomes equivalent to its strength, as defined by the Mohr–Coulomb strength criterion inputs. The block strength will decay to the residual level after the plastic (i.e., permanent) shear strain in the block becomes equivalent to the critical shear strain input parameter. Therefore, for BBMs with inelastic blocks, the overall deformation of the model is controlled by the formation of grain-scale fractures along contacts as well as the inelastic (i.e., permanent) deformation of blocks.

This study uses calibrated BBMs to simulate intact rock, and then joints were added to these models to test the predictive capabilities of the model. Only two-dimensional models were considered for this research due to simulation run-time limitations. Although three-dimensional BBMs have been developed in the literature, large block sizes are used in order to shorten the simulation run-times. Therefore, such BBMs do not approximate the grain structure of the rock being was modeled [40,48,49,50].

## 3. Blanco Mera Granite: Previous Laboratory Testing and Numerical Modeling

Ref. [24] presented the first study to test laboratory-scale rockmass analog specimens containing more than one degree of jointing with more than one joint set. Additionally, their study was relatively unique in that the analog specimens prepared were composed of real rock. This is in contrast to most other laboratory-scale rockmass analog studies using non-rock materials like plaster and gypsum [8,9,51,52], which tend to exhibit less brittle damage mechanisms than real rock [51,53].

In [24], compression tests were performed on specimens of Blanco Mera granite containing smooth joints with two different geometries: (1) one sub-vertical joint with two sub-horizontal joints and (2) two sub-vertical joints with three sub-horizontal joints. Trends were analyzed regarding the specimens’ elastic moduli, peak and residual strengths, Mohr–Coulomb and Hoek–Brown strength envelopes, and Geological Strength Index (GSI) values. The data from these experiments were later analyzed by [54] for trends in pre-peak damage thresholds and post-peak dilatancy. The relevant data from these two studies [24,54] are summarized in the following subsections.

### 3.1. Petrology

Blanco Mera is a coarse-grained granite from Spain [11] containing predominantly quartz, plagioclase, alkali feldspar, and mica mineral grains. Petrographic analysis was performed on the rock by [55]. Refer to Table A1 in Appendix A for a summary of the constituent mineral percentages in Blanco Mera granite and their grain sizes.

### 3.2. Mechanical Behavior of Intact Specimens

Blanco Mera granite has been thoroughly tested in the laboratory [11,24,54,55]. The data from [24,54] were used as the basis for model calibration in this study, as they contain the most complete data set. Refer to Table A2 in Appendix A for a summary of the mechanical properties of intact Blanco Mera granite.

### 3.3. Jointed Specimens

**Figure 1:**
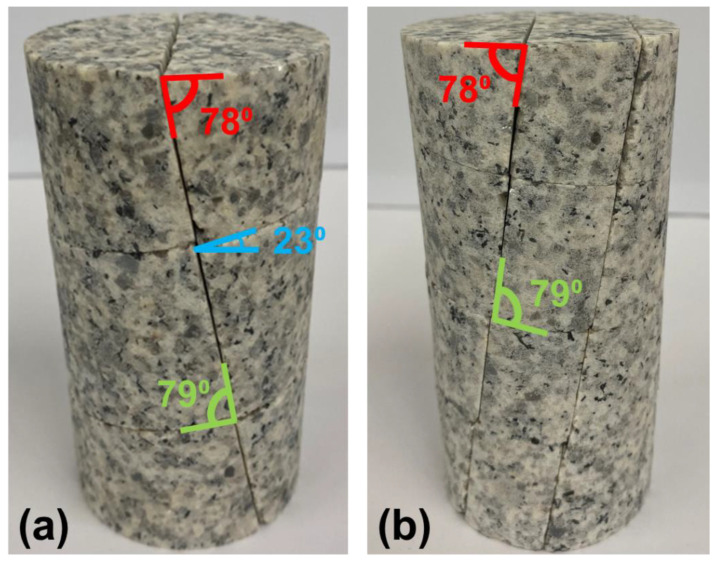
Images of the two jointed specimen types tested in the laboratory: (**a**) 1 sub-vertical joint and 2 sub-horizontal joints (1 + 2) and (**b**) 2 sub-vertical joints and 3 sub-horizontal joints (2 + 3). The dip of the sub-vertical joints is labeled in red, sub-horizontal joints is labeled in blue, and the angles between the two joint sets are labeled in green. Note that all joints are fully persistent. Photos courtesy: Dr. Leandro Alejano, University of Vigo.

Two specimen types representing two different pre-existing fracture network densities were tested by [24]: the 1 + 2 series, which contains 1 sub-vertical joint and 2 sub-horizontal joints (see Figure 1a) and the 2 + 3 series, which contains 2 sub-vertical joints and 3 sub-horizontal joints (see Figure 1b). All jointed were sawed into the granite at standardized locations. The sub-vertical joints are oriented 78 degrees from the horizontal and the sub-horizontal joints are oriented 23 degrees from the horizontal, as indicated in Figure 1. The joints were found to have a friction angle of approximately 30 degrees per tilt testing on the specimens. The confinement levels used for the triaxial tests on jointed specimens were 0.5, 1, 2, 4, 6, 10, and 12 MPa [24].

Figure 1 shows specimens with the joints oriented such that the angle between the sub-vertical and sub-horizontal joints is approximately 79 degrees. An alternative geometry of the 2 + 3 jointed specimens were also tested such that the angle between the two joint sets was 55 degrees (i.e., both the sub-vertical and sub-horizontal joint sets dipped in the same direction, rather than opposite directions as in Figure 1b). The authors of [24] found no notable difference in mechanical behavior between the two types of 2 + 3 jointed specimens.

### 3.4. Mechanical Behavior of Jointed Specimens

**Figure 2:**
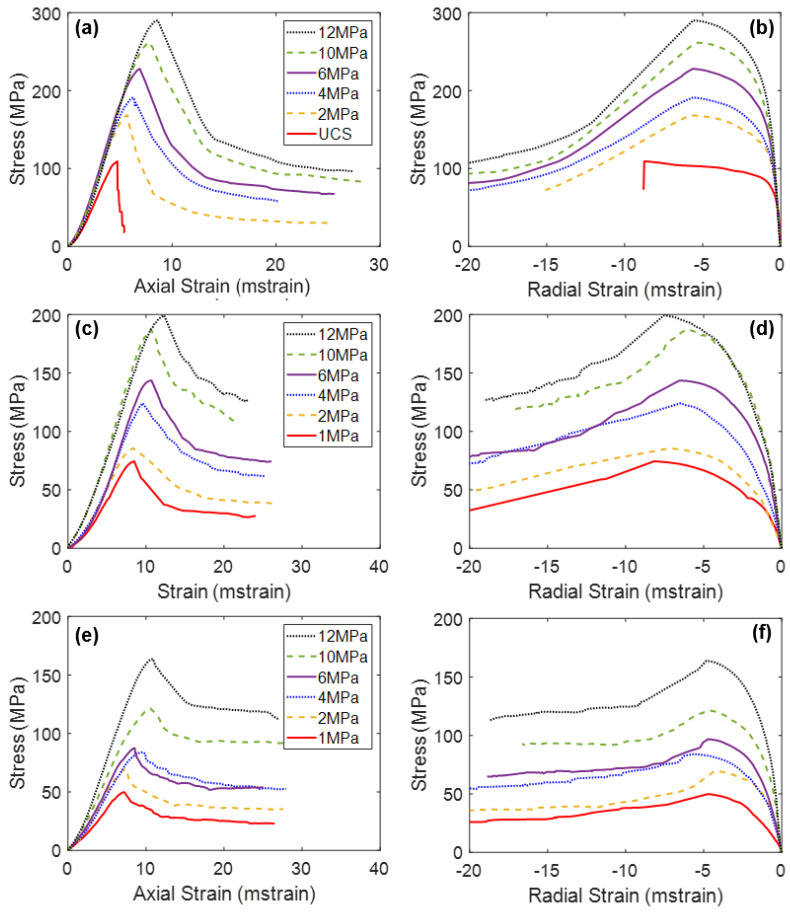
Average stress–axial strain curves (**a**,**c**,**e**) and average stress–radial strain curves (**b**,**d**,**f**) of Blanco Mera granite under various confinement levels, as tested in the laboratory [24]. Graphs (**a**,**b**) show intact specimen behavior, (**c**,**d**) show 1 + 2 specimen behavior, and (**e**,**f**) show 2 + 3 specimen behavior.

The results of the triaxial tests performed on the jointed specimens demonstrated that the presence of pre-existing joints notably influenced the mechanical behavior of the rock (see Figure 2). The results of the previous studies on Blanco Mera granite found an increase in jointing resulted in the following changes to mechanical properties, under all confinement levels:

- Decrease in Young’s modulus;
- Increase in Poisson’s ratio;
- Decrease in peak strength;
- Decrease in CI and CD values;
- No change in residual strength;
- Increase in apparent ductility;
- Decrease in Peak Dilation Angle;
- No change in Post-Peak Dilatancy at large strains.

More discussion on these trends can be found in [24,54].

### 3.5. Previously Calibrated Numerical Models of Jointed Blanco Mera Granite

Multiple numerical models have been developed to simulate the mechanical behavior of the jointed rockmass analog laboratory specimens tested by [24]. However, none of these studies have replicated all the mechanical attributes observed in the laboratory (see Table 1).

Each of these prior studies have one common aspect of their calibration process: a single calibration was performed to identify parameters that could replicate both the intact and jointed specimen behavior. Ref. [34] has noted the issue with using this sort of model for prediction purposes, where the model is calibrated to all known data without verification of the failure mode. For such models, its predictive capabilities outside the condition of calibration (i.e., for different rockmass conditions) are uncertain [56].

**Table 1:** Previous studies that aimed to model the rockmass analog laboratory specimens with smooth pre-existing joints from [24]. E = Young’s Modulus and v = Poisson’s Ratio. CI = Crack Initiation Stress and CD = Crack Damage Stress [57,58,59]. BPM = Bonded Particle Model per [60,61].

		----------------------Calibrated Material Attributes----------------------						
Study	Model Type	E	*v*	CI/CD	Peak Strength	Dilatancy	Residual Strength	Calibrated to Jointed Data?
[17]	LS-SRM (BPM)	x			x		x	x
[18]	BBM	x *			x			x
[19]	BPM	x			x		x **	x
[31]	BBM	x *			x			x
[32]	BPM	x *	x		x			x
[33]	Discrete: based on zero thickness interface elements	x			x		x	x
	Continuum: based on a multilaminate model	x			x	x	x	x

* Calibrated to secant Young’s Modulus rather than tangent Young’s Modulus. ** Did not match residual strength under higher confinement levels.

With all this in mind, this study has aimed to develop Bonded Block Models of the Blanco Mera granite specimens tested by [24] that accurately match the grain structure of the real rock, and is calibrated to match material attributes (i.e., stiffness and strength) as well as the micromechanical failure mechanism, as characterized by CI, CD, and post-peak stress–strain behavior. This study has also used forward simulation to predict the behavior of the jointed rockmass analog specimens. This process entails first back-calibrating an intact BBM (e.g., with no pre-existing joints) to laboratory data, including the micromechanical failure processes. Next, pre-existing joints are added to the model and no further calibration is performed. The degree of agreement (or disagreement) between the simulated predictions for the jointed specimen mechanical behavior and the observed behavior is representative of the predictive capabilities of the model.

## 4. Methods: Bonded Block Model (BBM) Development

A Bonded Block Model (BBM) with heterogeneous block and contact properties (by mineral type) was the chosen model type used in this study due to its ability to replicate the micromechanical behavior of real rock [28]. Details of BBM generation and calibration are included in the subsequent subsections.

### 4.1. Voronoi Generation

**Figure 3:**
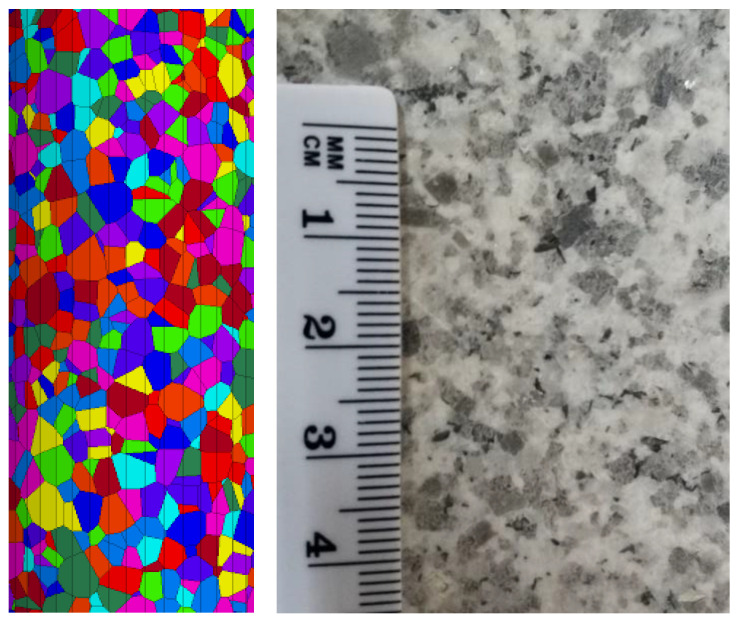
Neper-generated geometry (**left**) vs. real Blanco Mera granite (**right**) with approximately the same scale. The color-coding of the grains within the Neper model is arbitrary. Images from: [37].

A three-dimensional (3D) Bonded Block Model (BBM) of intact Blanco Mera granite was generated using the software Neper Polycrystal v3.5 [62]. The blocks were stochastically generated using input parameters of mean diameter, standard deviation of diameter, average sphericity, and standard deviation derived from petrographic analysis of real Blanco Mera granite specimens [63]. Diameter inputs in Neper Polycrystal were determined using a statistical method that incorporated estimated standard deviations of grain size diameters for each constituent mineral in Blanco Mera granite (refer to [37] for more details on this process). A variety of sphericity values and their standard deviations were tested to obtain an approximate visual match with the actual shapes of grains within Blanco Mera granite, where the grains of the model are relatively round like the real grains of Blanco Mera granite, but many of the smaller grains are more rectangular in shape, which is typical for micas. Figure 3 shows the BBM generated in Neper (trimmed to scale) beside a photograph of real Blanco Mera granite for comparison.

Two-dimensional (2D) cross sections of the 3D Neper model were generated using 3DEC. AutoCAD was used to prepare these cross sections to be built in UDEC v6, the software used to simulate compression tests.

### 4.2. Block Assignment to Mineral Groups

After the 2D Voronoi structure was built in UDEC, each block was assigned to a mineral group. A statistical method was used to assign blocks to mineral groups to achieve similar grain size distributions for each individual mineral in the model as in the real rock (see [37] for more detail). A comparison of the sizes and proportions of blocks representing different mineral groups within the BBM against those of the real Blanco Mera granite is presented in Table 2.

### 4.3. BBM with Pre-Existing Joints

**Figure 4:**
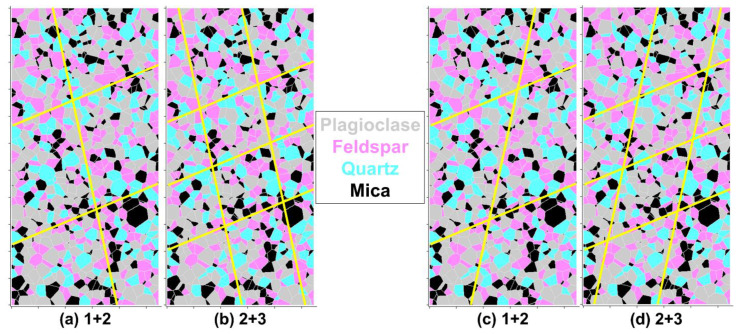
Bonded Block Models (BBMs) of Blanco Mera granite with the two joint patterns: (**a**,**c**) 1 + 2 and (**b**,**d**) 2 + 3. (**a**,**b**) are the main joint orientation geometry and (**c**,**d**) are the opposite joint orientation geometry. Joints are smooth, like in the corresponding laboratory specimens (see Figure 1).

In addition to the generation and calibration of an intact BBM of Blanco Mera granite, this study generated BBMs in UDEC with pre-existing smooth joints similar to the rockmass analog specimens tested in the laboratory (see Section 3.3). The exact locations of the joints corresponding to the 1 + 2 (Figure 4a) and the 2 + 3 (Figure 4b) joint patterns were determined by visually approximating their locations on the laboratory specimens (Figure 1). As discussed in Section 3.3, some jointed laboratory specimens were also tested with an opposite joint orientation. Although [24] found no difference in the strengths of these two specimen geometries, for completeness, the present study has also generated BBMs with the opposite joint geometry for mechanical behavior analysis (see Figure 4c,d).

**Figure 5:**
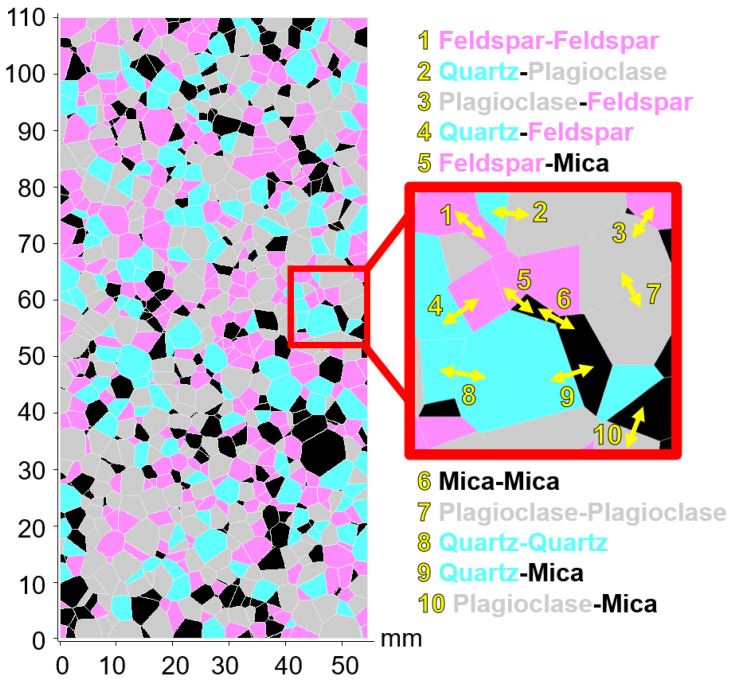
The BBM of Blanco Mera granite used in this study. It contains four mineral (block) types: alkali feldspar (pink), quartz (blue), plagioclase (gray), and mica (black). Ten unique contact types exist, labeled in yellow. Figure from: [64].

Once each block was assigned to one of the four mineral groups (plagioclase, quartz, alkali feldspar, or mica), input parameters were assigned to each block, as well as each corresponding contact, as depicted in Figure 5.

### 4.4. Compression Test Simulation

Two-dimensional UCS and triaxial test simulations were performed using the intact and jointed BBMs. All blocks were zoned with a dense mesh. Even the smallest blocks (0.005 mm diameter) were meshed with at least three distinct zones. A boundary condition restricting movement in the vertical direction was applied to the bottom of the model, which represents the base platen used in real laboratory. A constant velocity boundary condition of 0.01 m/s was applied to the top of the model in the vertical direction to simulate quasi-static loading (note that this value is consistent with previous numerical studies and cannot be directly compared to loading velocities used in physical laboratory tests [31,65,66]). A uniformly distributed horizontal load was applied to the lateral boundaries to simulate confining pressure. These boundary conditions represent the principal stress directions (the first principal stress is the axial loading, and the third principal stress is the lateral confining pressure). No boundary conditions were applied to the horizontal velocity stress at the top and bottom of the model or the vertical velocity or stress at the sides of the model, consistent with the actual conditions associated with compression testing.

During simulations, stress, axial strain, lateral strain, and the number of contacts failing in tension and shear were tracked to monitor macroscopic properties of the stress–strain behavior of the BBM. In the case of the inelastic block BBM, the number of zones yielding in tension and shear were also monitored. Stress was determined by calculating the average internal stress value within each zone of the BBM. Strains were calculated by computing the average displacement of the edge points of the BBM (in both the axial and lateral directions) from their original positions and then normalizing to the original corresponding dimension of the specimen.

### 4.5. Evaluation of Model Outputs

The macroscopic mechanical behavior of each BBM from the beginning of loading through peak strength was characterized using the following six material attributes for all confining stresses evaluated: Young’s modulus, Poisson’s ratio, Crack Initiation (CI) and Crack Damage (CD) parameters, peak strength, and peak dilation angle. The definition of each attribute, including references with further details on specific methods for estimation of values, are summarized in Table 3. The attributes were determined for the BBM using the stress and strain data tracked during each simulation. In addition to these properties, the stress–strain behavior post-failure was qualitatively assessed by visual assessment of stress–strain plots. Evaluation of these attributes for each model and the comparison with the corresponding laboratory data is part of the BBM calibration process (described in Section 4.7).

### 4.6. Hoek–Brown Fits

For the strength parameters discussed in Section 4.5, a model was fit to the data as a function of confinement using the Hoek–Brown failure envelope (Equation (1)).

(1) $$\sigma_{1}=\sigma_{3}+\sigma_{ci}\times {\left(m\times \frac{\sigma_{3}}{\sigma_{ci}}+s\right)}^{a}$$

where *σ*_1_ is the maximum effective principal stress at a given strength threshold (either peak strength, CI, or CD), *σ*_3_ is the minimum effective principal stress (confinement), *σ_ci_* is the value of the given strength threshold (peak strength, CI, or CD) of the intact rock under unconfined conditions, *m* is the Hoek–Brown material parameter, and *s* and *a* are rockmass characteristic parameters.

For the intact specimen data, *s* was set equal to 1 and *a* was set equal to 0.5 [67]. *σ_ci_* and *m* were fit to stress data (*σ*_1_) as a function of confinement (*σ*_3_) using a least-squares fitting method. For the jointed models (1 + 2 and 2 + 3), *σ_ci_* was set equal to that of the intact rock and *s*, *m*, and *a* were allowed to independently vary. All four Hoek–Brown fit parameters are reported along with the results in Section 5 and Section 6.

**Table 3:** Summary of the material attributes used to quantify the mechanical behavior of the specimens in this study. The definition, method of determination, and references are included for each attribute.

Material Attribute	Method of Determination	References
Young’s Modulus	The slope of the axial stress–axial strain curve within the linear elastic portions of the axial strain data	[68]
Poisson’s Ratio	The slope of the linear elastic portion of the lateral strain–axial strain curve	[69]
Crack Initiation (CI) Threshold	The point of non-linearity in the lateral strain–axial strain or lateral strain–axial stress plot	[49,57,70,71]
Crack Damage (CD) Threshold	The point of non-linearity in the axial stress–axial strain plot	[49,57,70]
Peak Strength	The largest stress achieved	-
Peak Dilation Angle	Maximum value of the dilation angle, where dilation angle varies as a function of plastic shear strain and confinement	[54,72,73]
Brittleness	Slope of post-peak strength decrease; high rate = an increased slope indicates more brittle behavior	[74,75]

### 4.7. Model Calibration to Intact Specimen Laboratory Data

Both the elastic block and inelastic block BBMs were calibrated by iteratively adjusting their input parameters. To ensure that the model’s behavior matched laboratory data under the full range of confinement levels in which data are available, the behavior of the BBM under unconfined and confined conditions with 12 MPa of confinement were analyzed for each input parameter set used during the calibration process. Once the BBM was calibrated to match the laboratory data of the lower and upper bounds of confinements, the BBM was run under intermediate confinement levels to evaluate the match to all available laboratory data under each confinement level.

Although most of the model input parameters cannot be determined directly from laboratory testing or field measurements, a reasonable range of values for each input parameter can be constrained based on the results of previous studies using BBMs. Starting input parameters were selected from the literature, and efforts were made to avoid making large changes to any individual input parameter.

Each time a model was run with a new set of input parameters, the mechanical attributes described in Section 4.5 were determined and compared to laboratory data [24,54]. Once the emergent behavior matched that of the intact specimen laboratory data for Blanco Mera granite, the calibration was deemed complete. The procedures followed for calibration of both the elastic and inelastic block BBMs are documented in greater detail in the following subsections.

The elastic block BBM was calibrated first, as it requires all the same inputs as inelastic block BBMs other than block yield and dilation parameters. Therefore, the elastic block and contact input parameters calibrated during elastic block BBM calibration were used as the starting point for the inelastic block BBM calibration (more on this in Section 4.7.2).

#### 4.7.1. Elastic Block BBM Calibration

For the elastic block BBM calibration, Young’s Modulus, Poisson’s Ratio, CI, CD, and peak strength were matched to laboratory data. No attempt was made to reproduce post-peak attributes, as post-peak behavior cannot be matched using elastic blocks. Elastic block BBMs neglect the intra-granular fracturing process, which is an important damage mechanism in the post-peak region of stress–strain curves, as well as for stress–strain curves under higher confinement levels [28].

Since the Blanco Mera BBM is heterogeneous, meaning that there is more than one type of block (corresponding to the four mineral groups) and more than one type of contact (see Figure 5), each type of block and contact can be assigned different values as input parameters. Therefore, an input parameter can be varied in the BBM by changing each type’s value by the same amount so that the average value changes, or by changing each type’s values systematically so that the spread of values changes (i.e., change in heterogeneity). Refer to [64] for an in depth parametric study on BBM input parameter heterogeneity.

A summary of the elastic block BBM calibration process for Blanco Mera granite in sequential order is as follows:

1. **Poisson’s Ratio**: Macroscopic Poisson’s Ratio is controlled by the block elastic moduli and ratio of contact shear stiffness (jks) to contact normal stiffness (jkn). Using starting block moduli values from other BBMs containing grains of the same mineral types, a Poisson’s Ratio of 0.17 (see Table A2) could not be attained without modifying the jks to jkn ratio to be outside the range reported by previous studies using elastic block BBMs. Therefore, the jks to jkn ratio was set to 0.65, which is towards the upper bound of what has been used in previous studies. Instead, the block moduli were lowered until the correct Poisson’s Ratio was achieved.
2. **Young’s Modulus**: jks and jkn values were increased proportionally to maintain the same ratio until the BBM replicated the macroscopic Young’s Modulus.
3. **Crack Initiation (CI)**: CI corresponds to the onset of tensile fracturing, so this property is predominantly controlled by the tensile strength of block contacts. Increasing the contact tensile strength increases the value of CI under all confinements. Additionally, the overall heterogeneity of the BBM (in terms of both blocks and contacts) can be increased to decrease the value of CI [64].
4. **Unconfined Peak Strength**: UCS is primarily controlled by contact peak cohesion of contacts where increasing peak cohesion increases the UCS of the BBM. Ref. [64] also found that increasing the heterogeneity of contact peak cohesion decreased the BBM’s UCS.
5. **Crack Damage (CD)**: Since CD corresponds to the onset of shear fracturing, increasing the shear strength of the contacts increases the CD value of the BBM. Therefore, CD is controlled by contact peak cohesion and peak friction angle, where contact peak cohesion has a greater influence on the unconfined CD and peak friction angle has a greater influence on the confined CD. Additionally, both the heterogeneity of contact peak cohesion and the overall heterogeneity of the BBM (in terms of both block and contact properties) can be increased to decrease the value of CD [64].
6. **Confined Peak Strength**: Peak and residual contact friction angles predominately control the confined strength of the BBM. It should be noted that increasing the peak friction angle increases the confined peak strength but will also increase the CD of the BBM. On the other hand, increasing contact residual friction angle increases the confined peak strength of the BBM, but does not notably affect the model’s CD.

The calibrated block mineral group and contact input parameters are included in Table 4 and Table 5, respectively.

#### 4.7.2. Inelastic Block BBM Calibration

Inelastic block BBMs require all the same inputs as elastic block BBMs, but also require block strength properties. The starting inelastic block parameters were taken from a previously calibrated BBM of Creighton granite by [28]. The same set of inelastic parameter values was assigned to all block types (i.e., mineral types), despite each type having unique elastic moduli values, as it has previously been determined that heterogeneous inelastic block parameters are not necessary to match all mechanical attributes at the laboratory-scale [28]. Additionally, assigning each block type different values of inelastic block input parameters would significantly increase the number of inputs in the model, making the calibration process more difficult and increasing the potential for model non-uniqueness.

The iterative process of inelastic block BBM calibration by trial-and-error is identical to that of the elastic block BBM calibration but involves more input parameters. Unlike elastic block BBMs, inelastic block BBMs are able to match the post-peak behavior of rock, where the inelastic block input parameters influence the post-peak behavior. In fact, for the range of input parameter values tested during this calibration process, the inelastic block parameters were not found to have a notable effect on pre-peak mechanical attributes of the BBM (Young’s Modulus, Poisson’s Ratio, CI, or CD). Rather, these input parameters only affected the peak strength, and post-peak behavior. Additionally, the elastic block and contact input parameters controlled the pre-peak mechanical attributes in the same way as summarized in Section 4.7.1. Prior to final model calibration, Ref. [76] documented the results of a sensitivity analysis evaluating the influences of inelastic block input parameter on the peak strength and post-peak behavior of the Blanco Mera inelastic block BBM, which are summarized in Table A3 in Appendix A.

During the inelastic block BBM calibration process, emphasis was placed on the inelastic block input parameters rather than the contact input parameters. However, contact input parameters were also varied in certain cases where the inelastic block input parameters did not induce a large enough change on the model behavior. In particular, contact residual friction angle and dilation angle were found to have an effect on the post-peak behavior of the BBM, so these input parameters were frequently changed during the calibration process in order to match the BBM’s post-peak brittleness to laboratory data.

The final values calibrated for the inelastic block BBM of Blanco Mera granite are listed in Table 4 (block elastic moduli), Table 6 (block inelastic parameters), and Table 7 (contact parameters).

### 4.8. Jointed Model Input Parameters

After the intact BBM was calibrated for both the elastic and inelastic block cases, smooth pre-existing joints were added to the BBM in the same orientations as those in the laboratory tests (see Section 4.3). As discussed previously, the input values for the pre-existing joints were not calibrated but rather estimated from similar models in the literature or taken directly from laboratory data. The pre-existing joints were modeled using an elastic-plastic constitutive model with Coulomb slip. With this constitutive model, the strength of the joints is governed by the peak friction angle and does not decay to a residual level with slip [47]. The cohesion and tensile strength of a pre-existing smooth joint with no infilling are zero. Therefore, the required input parameters of the pre-existing joints are: jkn, jks, cohesion, friction angle, and tensile strength. The values used for these inputs are listed in Table 8.

The jks and jkn values were taken from a previous study that modeled intact and jointed Blanco Mera granite [19], as these values were within the middle of the range of values used in all of the previous studies that modeled jointed Blanco Mera granite and had comparable modeling methods including other BBMs [18,31] or Bonded Particle Models [19]. A small sensitivity analysis was performed on the persistent joint jkn and jks values for one of the 1 + 2 jointed BBMs. Stiffness values were varied from the values listed in Table 8 to analyze the effect that joint stiffness values had on the model strength. Increasing jkn from 500 to 1000 GPa/m and jks from 50 to 100 GPa/m resulted in an increase in model strength by 10% under low confinement (1 MPa) and an increase in model strength by 2% under high confinement (12 MPa). Although very large changes in stiffness value magnitude (on the order of multiple magnitudes) are expected to notably affect the model’s strength, such values are outside the range of those used by previous studies and are not reasonable values to use for such joints.

A friction angle of 30 degrees was determined based on the basic friction angle of joints as determined in the laboratory [24]. Note that no calibration was performed for the jointed BBMs; all joint parameter values were estimated ahead of time based on values documented in the literature, as might be the case in practical scenarios where a model is being used to make forward predictions.

## 5. Intact BBM Calibration Results and Discussion

### 5.1. Mechanical Attributes Derived from Stress–Strain Curves

**Figure 6:**
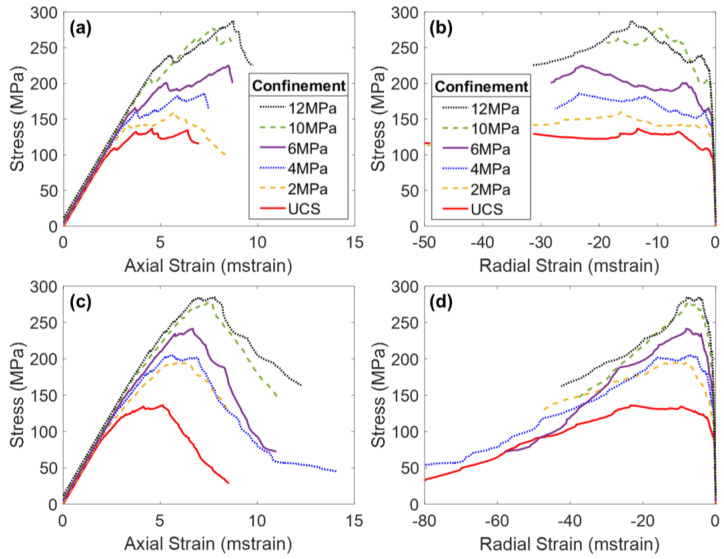
Axial stress–axial strain (**a**,**c**) and axial stress–radial strain (**b**,**d**) curves of the intact elastic block BBM (**a**,**b**) and the inelastic block BBM (**c**,**d**). Strain is plotted in milli-strain (mstrain).

Figure 6 presents the stress–strain curves of the two intact BBMs (elastic and inelastic blocks) under various confinement levels. Note on Figure 6a,c that all simulations exhibited the same Young’s modulus regardless of the confining pressure. This trend is not consistent with laboratory experiments (see Figure 2), which show an increasing Young’s modulus with increasing confining pressure [24,54,55]. However, due to the inherent nature of Voronoi tessellations, BBMs do not contain pre-existing discontinuities that are responsible for the artificial reduction in the stiffness of the low-confinement laboratory specimens.

**Figure 7:**
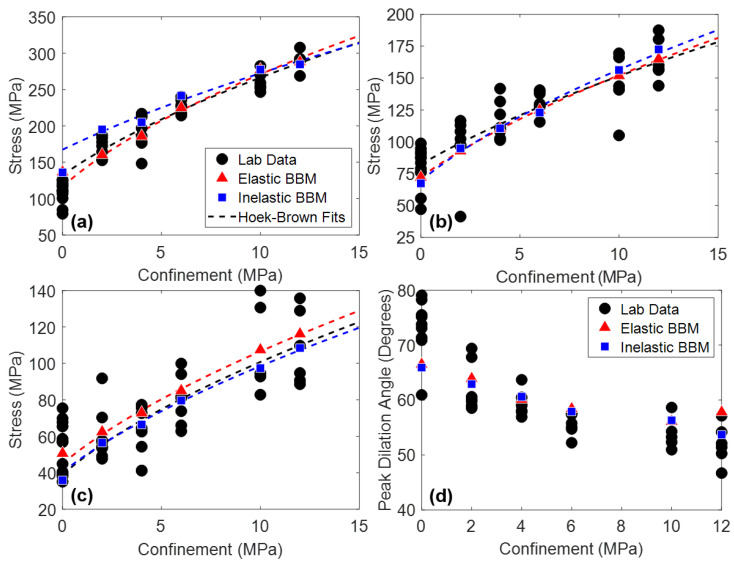
(**a**) Peak strength, (**b**) CD, (**c**) CI, and (**d**) peak dilation angle of laboratory data (black circles), the elastic block BBM (red triangles), and the inelastic block BBM (blue squares) of Blanco Mera granite. Hoek–Brown fits are included where applicable. Note that peak dilation angle data were not reported in [54] but were manually interpreted from the strain data provided.

In addition to Young’s modulus and Poisson’s ratio, peak strength, CI, CD, and peak dilation angle in both model types were matched to laboratory data, the results of which are shown in Figure 7. The Hoek–Brown best fit parameters corresponding to the curves plotted on Figure 7 are included in Table A4 in Appendix A.

Note that the calibration target for the strength of the unconfined BBMs (both with elastic and inelastic blocks) was at the upper end of the range of laboratory data. Many data points of unconfined strength are believed to be artificially low due to the presence of small pre-existing fractures in the specimens, which are not modeled in the BBM. These pre-existing fractures do not notably affect the strength of specimens tested under confinement because the confining pressure closes these fractures prior to loading.

Considering the material attributes discussed so far (Young’s modulus, Poisson’s ratio, CI, CD, peak strength, and peak dilation angle), both the elastic and inelastic block BBMs are virtually identical. One exception is the peak strength envelope of the inelastic block BBM. Although both the elastic and inelastic block BBMs were calibrated to the same values of peak strength under no confinement and under 12 MPa of confinement, the intermediate confinement simulations resulted in different peak strength values (see Figure 7a). This is expected, as the elastic and inelastic block BBMs behave differently at the grain-scale (e.g., inelastic blocks incur permanent deformation whereas elastic blocks do not). The peak strength of the inelastic block BBM is larger than that of the elastic block BBM under confinement levels of 2 MPa to 6 MPa. At larger confinements (10+ MPa), the peak strengths of the two BBMs are virtually the same.

The main difference between the stress–strain results of the two BBMs occurs between CD and peak strength, and into the post-peak. The elastic block BBM (Figure 6a) exhibits significant strain hardening, where the peak strength is not reached until large values of axial strain. Large amounts of strain hardening are commonly associated with elastic block BBMs [25,28,66]. This amount of strain hardening does not exist for the inelastic block BBM (see Figure 6c). As intragranular damage (zone yield) becomes more prevalent in the model after CD, the strength of the model is reduced. The internal deformation of blocks helps to decrease friction that is mobilized in the BBM as large sections of intact material break off from one another along failed contacts, which decreases the extent of strain hardening. The behavior of the inelastic block BBM is akin to that of the real Blanco Mera granite, which is brittle in behavior and does not exhibit so much strain hardening (see Figure 2). The post-peak brittleness of the inelastic block BBM (see Figure 6c) is realistic, where the unconfined simulation was relatively brittle (i.e., had a sharp drop in strength following the peak strength) and the sharpness of the drop in strength generally decreased with increasing confinement. On the other hand, the elastic block BBM did not exhibit brittle post-peak stress–strain behavior due to the previously described excessive strain hardening phenomenon.

### 5.2. Damage Mechanisms

**Figure 8:**
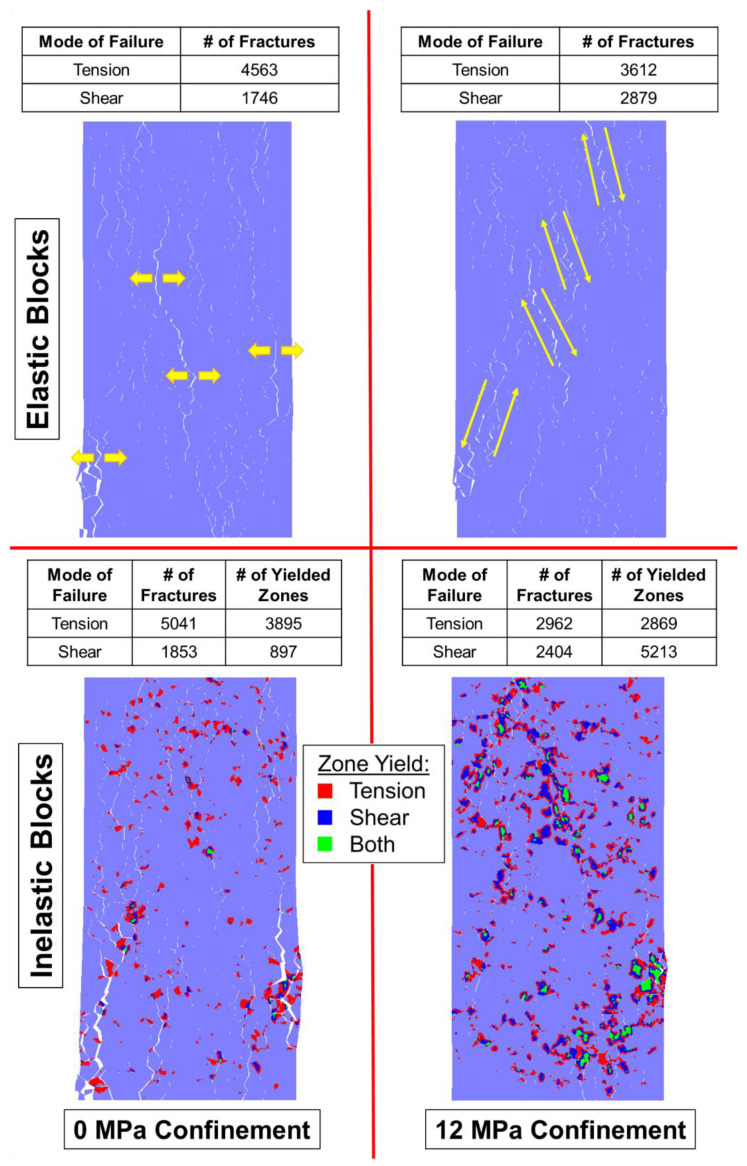
Intact BBMs at failure (peak strength) showing the location of macroscopic fractures with visible separation. At (**top left**) is the elastic block BBM under unconfined conditions, (**top right**) is the elastic block BBM under 12 MPa of confinement, (**bottom left**) is the inelastic block BBM under unconfined conditions, and the (**bottom right**) is the inelastic block BBM under 12 MPa of confinement. Inelastic models indicate the location of zones that have yielded. Yellow arrows are included to highlight the tensile fracturing (**top left**) and shear en echelon fracturing (**top right**).

As discussed in Section 4, the behavior of a BBM must replicate the micromechanical failure of real rock to be fully calibrated, as well as to be predictive. Brittle rock under low confinement tends to form tensile fractures, which dilates the specimen. Such rocks fail by axial splitting [77]. As the rock is placed under higher confinement levels, the formation of tensile fractures is suppressed, shear fractures become more prevalent, and less dilation occurs [78]. Such rocks fail by shear banding, which is a less brittle failure mechanism [75]. This switch from tensile fracture dominated failure to shear fracture dominated failure is captured by both the elastic and inelastic block BBMs, as indicated in Figure 8 where each model is shown at its peak strength.

Axial splitting occurs in both the elastic block and inelastic block BBMs under zero confinement conditions (indicated by the yellow arrows in the top left panel of Figure 8). There is large dilatancy caused by separation along the axially oriented fractures. On the other hand, under 12 MPa of confinement, less separation occurs along the fractures, which results in less dilation.

Although a macroscopic failure plane under higher confinements is not obvious in Figure 8, significant en echelon shearing has occurred (indicated by the yellow arrows in the top right panel of Figure 8) within both the elastic and inelastic block BBM, characterized by both shear fracture formation and, in the case of the inelastic block BBM, shear zone yield of the blocks. In the models under zero confinement, notably more tensile fractures (and tensile zone yield) exist compared to shear. In the models under 12 MPa of confinement, the number of tensile and shear fractures (and zone yield, in the case of the inelastic block BBM) are nearly equivalent. As such, the models are also behaving like real rock, where tensile fracture formation and propagation is suppressed by confinement and shear failure dominates.

Ref. [28]’s study on elastic and inelastic block BBMs previously found that inelastic block yield was more important for the replication of brittle rock behavior under higher confinement levels than under lower confinement levels. When comparing the elastic and inelastic block BBMs’ failure modes under 0 MPa of confinement (left panels on Figure 8), there is not a notable difference in the pervasive failure pathways between the two model types. However, when comparing the elastic and inelastic block BBMs’ failure modes under 12 MPa of confinement (right panels on Figure 8), the inelastic block BBM exhibited notably less fracture separation and macroscopic coalescence. Instead, the inelastic block yield contributed to the model’s pervasive failure pathways.

## 6. Jointed BBM Results and Discussion

### 6.1. Mechanical Attributes Derived from Stress–Strain Curves

**Figure 9:**
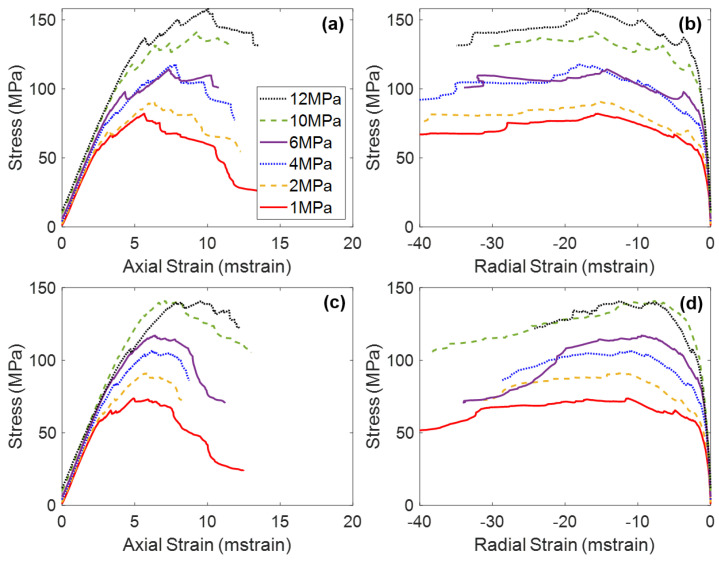
Axial stress–axial strain (**a**,**c**) and axial stress–radial strain (**b**,**d**) curves of the 1 + 2 elastic block BBM (**a**,**b**) and the inelastic block BBM (**c**,**d**). All curves correspond to the 1 + 2 jointed model geometry shown in Figure 4a. Strain is plotted in milli-strain (mstrain).

**Figure 10:**
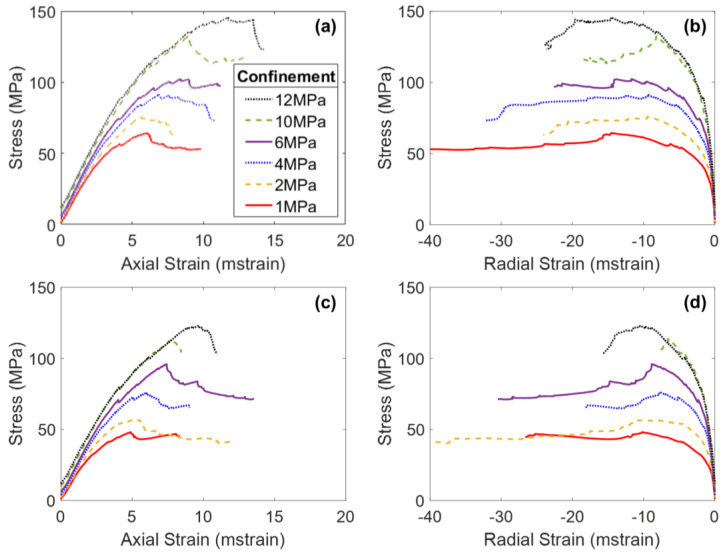
Axial stress–axial strain (**a**,**c**) and axial stress–radial strain (**b**,**d**) curves of the 2 + 3 elastic block BBM (**a**,**b**) and the inelastic block BBM (**c**,**d**). The curves on panels a and b correspond to the 2 + 3 jointed model shown in Figure 4b. The curves on panels c and d correspond to the 2 + 3 jointed model shown in Figure 4d. Strain is plotted in milli-strain (mstrain).

**Figure A1:**
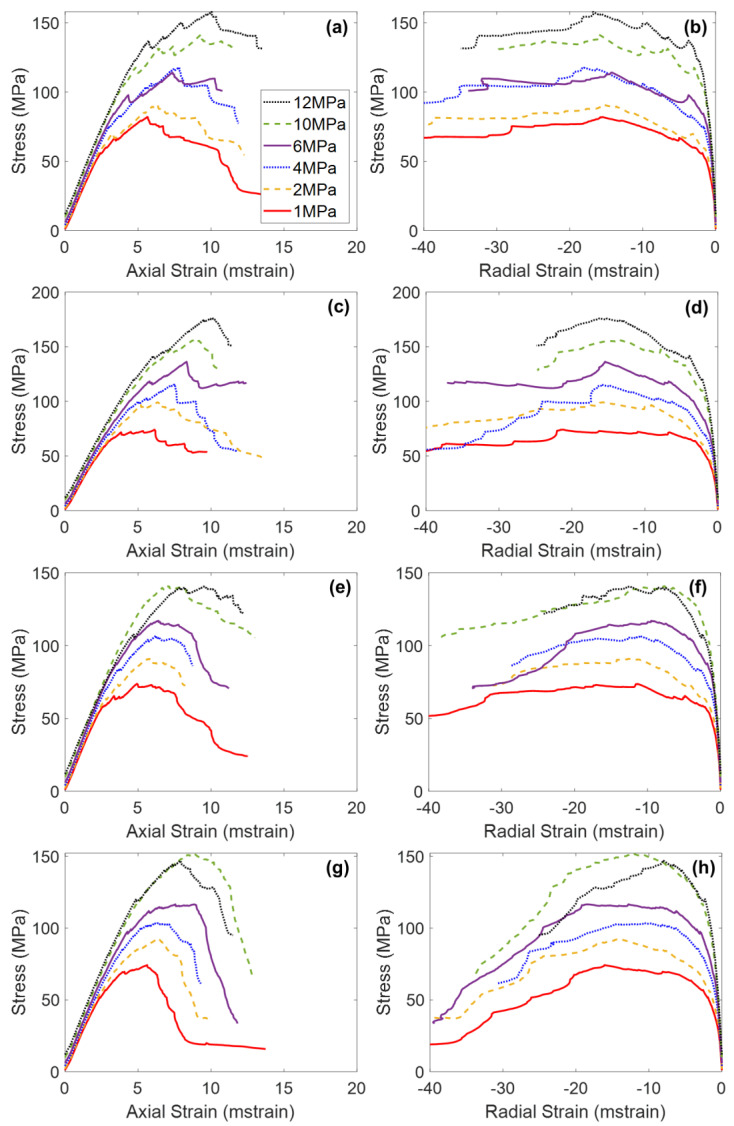
Axial stress–axial strain (**a**,**c**,**e**,**g**) and axial stress-radial strain (**b**,**d**,**f**,**h**) curves of the 1 + 2 elastic block BBM (**a**–**d**) and the inelastic block BBM (**e**–**h**). Strain is plotted in milli-strain (mstrain): (**a**–**d**) correspond to the model shown in Figure 4a (**e**–**h**) correspond to the model shown in Figure 4c.

**Figure A2:**
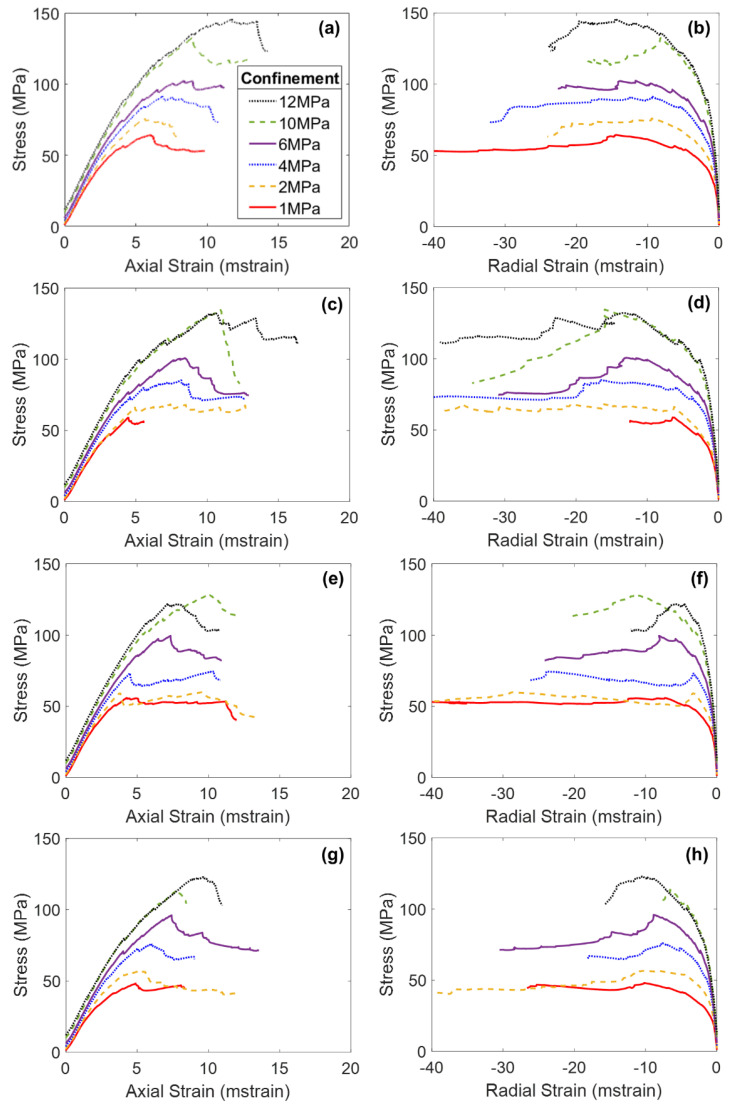
Axial stress–axial strain (**a**,**c**,**e**,**g**) and axial stress-radial strain (**b**,**d**,**f**,**h**) curves of the 2 + 3 elastic block BBM (**a**–**d**) and the inelastic block BBM (**e**–**h**). Strain is plotted in milli-strain (mstrain): (**a**–**d**) correspond to the model shown in Figure 4b (**e**–**h**) correspond to the model shown in Figure 4d.

The stress–strain results of the jointed BBMs are presented in Figure 9 and Figure 10 for the 1 + 2 and 2 + 3 models, respectively. The most representative set of stress–strain curves of each of the two jointed models (Figure 4) are included for simplicity. Both joint orientations were found to produce similar macroscopic stress–strain responses in the BBMs have been included as Figure A1 and Figure A2 in Appendix A, for the 1 + 2 and 2 + 3 jointed models, respectively.

**Figure 11:**
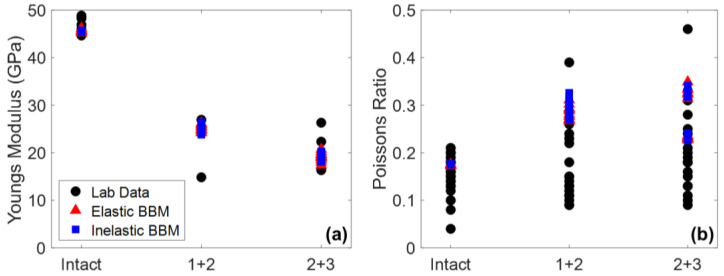
(**a**) Young’s modulus and (**b**) Poisson’s ratio of the elastic (red triangle) and inelastic (blue square) BBMs compared to laboratory data (black circles). The results intact, 1 + 2, and 2 + 3 series are included as well as both joint geometries (see Figure 4). Note that the laboratory Young’s modulus data only includes data under 12 MPa of confinement.

When comparing the elastic regions of the stress–strain curves of the intact BBM (Figure 6) to the jointed BBMs (Figure 9 and Figure 10), it is noted that the slope of this linear region of the stress–axial strain curves (i.e., Young’s modulus) decreases as a function of increasing jointing. This same trend was noted for the laboratory specimens as well (see Figure 2). The pre-existing joints in the jointed BBMs decrease the stiffness of the overall model. This effect becomes less extreme with increased confinement, which increases the normal stress on the joints and inhibits slip, which thus increases the model’s overall stiffness. The BBMs were able to match this decrease in Young’s modulus, as well as the slight increase in Poisson’s ratio as a function of increased jointing (see Figure 11). Note that the results of both joint geometries (see Figure 4) are included in Figure 11.

Both the elastic and inelastic block BBMs were able to predict the Young’s modulus and Poisson’s ratio of Blanco Mera granite equally well. The models with joint geometries shown in Figure 4c,d were found to have higher values of Poisson’s ratio than their geometric counterparts shown in Figure 4a,b. Although this difference in Poisson’s ratio was small for the 1 + 2 models, this difference is more noticeable for the 2 + 3 models, where the two groups in data points on Figure 11b correspond to the two different geometry types. However, the Poisson’s ratio values of both model joint geometries fall within the range of values of the laboratory specimens.

**Figure 12:**
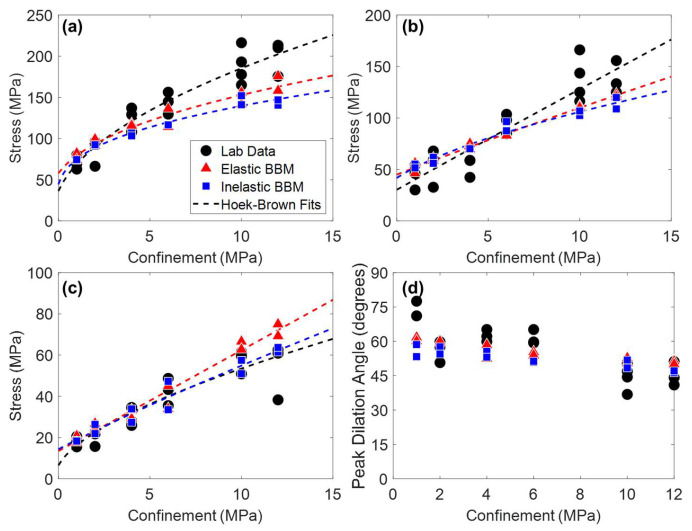
(**a**) Peak strength, (**b**) CD, (**c**) CI, and (**d**) peak dilation angle of laboratory data (black circles), the elastic block BBMs (red triangles), and the inelastic block BBMs (blue squares) of Blanco Mera granite containing the 1 + 2 joint pattern (see Figure 4). Hoek–Brown fits are included where applicable. The two BBM data points per confinement level correspond to the two joint orientations per Figure 4. Note that peak dilation angle data were not reported in [54] but were manually interpreted from the strain data provided.

**Figure 13:**
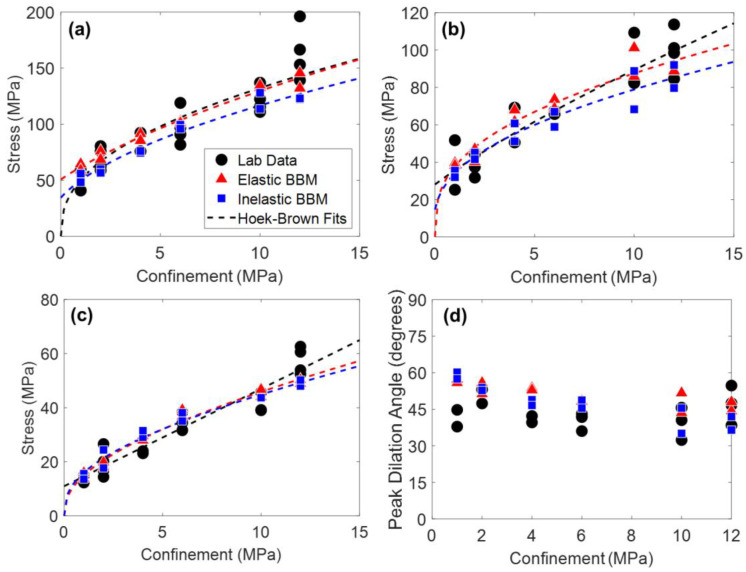
(**a**) Peak strength, (**b**) CD, (**c**) CI, and (**d**) peak dilation angle of laboratory data (black circles), the elastic block BBM (red triangles), and the inelastic block BBM (blue squares) of Blanco Mera granite containing the 2 + 3 joint pattern (see Figure 4). Hoek–Brown fits are included where applicable. The two BBM data points per confinement level correspond to the two joint orientations per Figure 4. Note that peak dilation angle data were not reported in [54] but were manually interpreted from the strain data provided.

Peak strength, CI, CD, and peak dilation angle values of models of both joint geometries are shown in Figure 12 and Figure 13 for the 1 + 2 and 2 + 3 joint cases, respectively. The Hoek–Brown best fit parameters corresponding to the curves plotted on Figure 12 and Figure 13 are included in Table A5 and Table A6 in Appendix A, respectively. No trend was observed regarding the effect of the jointed model geometry (see Figure 4) on the mechanical attributes plotted in Figure 12 and Figure 13. As such, data points of both model geometries are included in Figure 12 and Figure 13 and the Hoek–Brown best fit lines were created using all of the data.

Per Figure 12 and Figure 13, both the elastic and inelastic block BBMs were able to reasonably predict the behavior of the jointed laboratory specimens in terms of strength, cracking thresholds, and dilatancy. Although the exact values of each material attribute were not able to be predicted, the BBMs exhibited similar trends in values as a function of confinement (the Hoek–Brown fits). One exception is the 1 + 2 jointed specimens under higher confinement levels (10 MPa of confinement and higher). Both BBMs underpredicted the trend (i.e., the Hoek–Brown fit) of peak strength and CD by as much as 20% in the case of the elastic block BBM and 27% in the case of the inelastic block BBM.

Regarding the post-peak behavior, the inelastic block BBM was able to better replicate the increase in apparent post-peak ductility as a function of increased jointing (see Figure 6c, Figure 9c and Figure 10c for BBM curves and Figure 2a,c,e for the corresponding laboratory curves). Due to the excessive strain hardening of the elastic block BBM, this increase in apparent ductility as a function of increased jointing is not clear (see Figure 6a, Figure 9a and Figure 10a). As discussed in Section 5, inelastic blocks are required to match the post-peak behavior of brittle rock. Per Figure 7, inelastic blocks were also required to match the post-peak behavior of the jointed laboratory specimens. The stress–strain curves of the inelastic block BBM exhibit less strain hardening, which better matches laboratory data (see Figure 2).

Although the inelastic block BBM better predicts the post-peak behavior of Blanco Mera granite, elastic block BBMs require notably less run-time, which makes the elastic block BBM the most favorable for predictive purposes where accurately representing post-peak behavior is not necessary.

### 6.2. Damage Mechanisms

**Figure 14:**
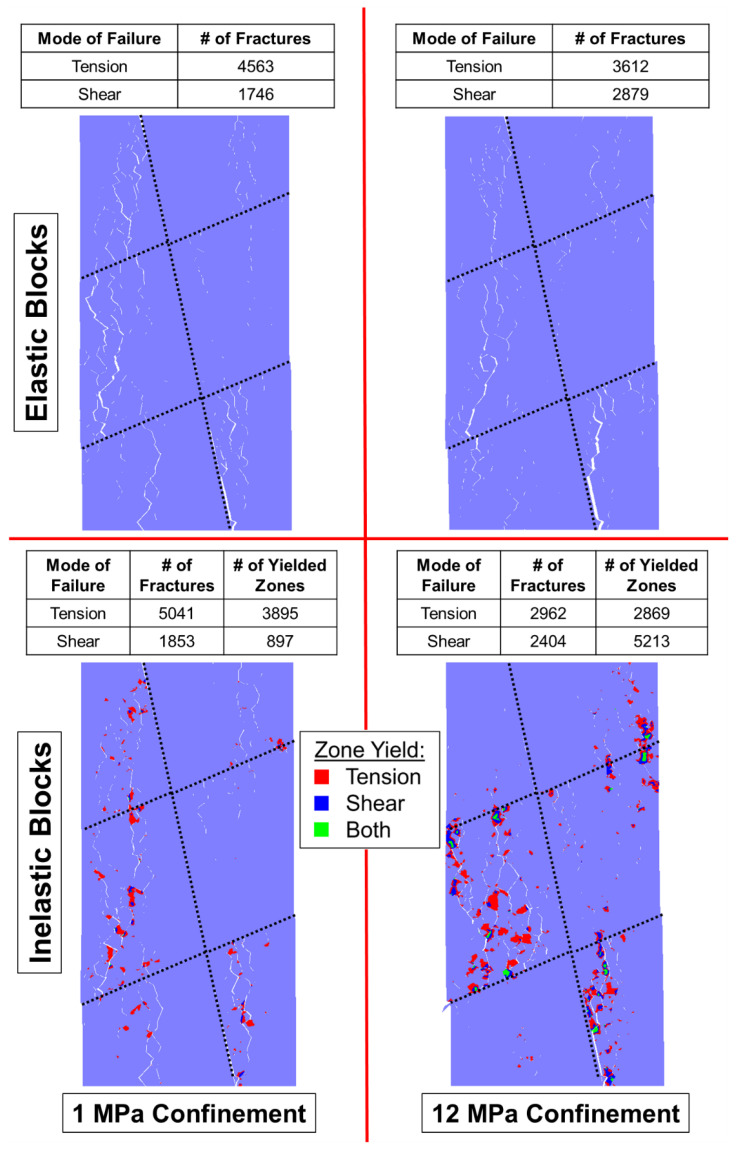
**The** 1 + 2 jointed BBMs at failure (peak strength) showing the location of macroscopic fractures with visible separation: (**top left**) is the elastic block BBM under unconfined conditions, (**top right**) is the elastic block BBM under 12 MPa of confinement, (**bottom left**) is the inelastic block BBM under unconfined conditions, and the (**bottom right**) is the inelastic block BBM under 12 MPa of confinement. Inelastic models indicate the location of zones that have yielded.

**Figure 15:**
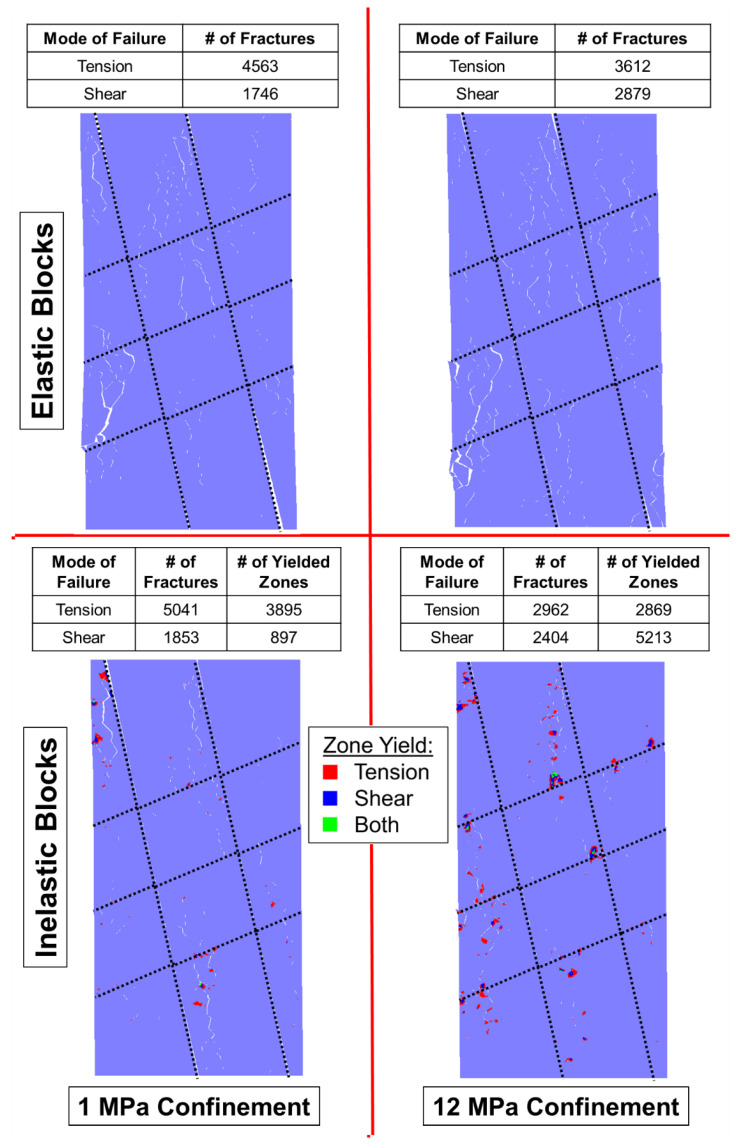
**The** 2 + 3 jointed BBMs at failure (peak strength) showing the location of macroscopic fractures with visible separation: (**top left**) is the elastic block BBM under unconfined conditions, (**top right**) is the elastic block BBM under 12 MPa of confinement, (**bottom left**) is the inelastic block BBM under unconfined conditions, and the (**bottom right**) is the inelastic block BBM under 12 MPa of confinement. Inelastic models indicate the location of zones that have yielded.

The presence of the pre-existing joints influenced the failure mechanisms of the laboratory specimens, where the rock failed via a mixed mode of intact brittle material failure and slip along the joints [24]. The intact brittle material fails via the same fracturing processes described in Section 5: material fails via axial splitting induced by the formation of tensile fracturing when the confinement level is low, and material fails via shear banding induced by the formation of shear fractures when the confinement level is high. However, the overall jointed specimens fail by both intact material failure as well as slip along the joints. The BBMs were able to replicate both the intact material failure, as well as slip along the pre-existing joints, as shown in Figure 14 and Figure 15 for the 1 + 2 and 2 + 3 jointed models, respectively.

Figure 14 shows the 1 + 2 jointed models failing largely through intact material failure, where the low confinement models exhibited axially oriented tensile fracturing and the high confinement models exhibited the same en echelon shear fracturing as noted in the intact BBMs (Figure 8 in Section 5). Also like the intact BBMs, the inelastic 1 + 2 jointed BBMs (bottom panels in Figure 14) exhibited zone yield in lieu of some of the contact fracturing seen in the elastic BBMs. The pervasive failure pathway in the inelastic models is composed of both fractures and zone yield.

Different trends are observed in Figure 15, which shows the 2 + 3 jointed models failing less through the formation of axial fracturing and more en echelon shearing than the intact and 1 + 2 models (Figure 8 and Figure 14, respectively), even under the low confinement level. This decrease in brittle failure as a function of increased jointing has been noted previously by [12] in the context of SRM and can also be observed from the Blanco Mera granite laboratory results from [24]. The 2 + 3 jointed models also do not exhibit large, pervasive failure pathways (formed by either fractures in the case of the elastic block models or a mixture of fractures and zone yield in the case of the inelastic block models). This indicates that the dominant mode of failure in these models is slip along the persistent joints. Therefore, slip along the persistent joints becomes a more important component of specimen failure as the degree of jointing increases.

## 7. Discussion and Implications for Future Work

The results of this study have quantitatively evaluated the concept of Synthetic Rockmass Modeling (SRM), where a DFN can be added to a calibrated discrete model of intact rock to simulate rockmass behavior. Ultimately, we found that a well-calibrated BBM can be used to predict the behavior of rock under jointing conditions that are different from those for which the BBM was calibrated (i.e., intact rock). This BBM of Blanco Mera granite was calibrated to intact laboratory data and its predictive capabilities were verified by adding smooth, persistent joints to the model and comparing the behavior of the jointed models to respective jointed laboratory specimen data. The BBM was able to replicate the behavior of the rock under these new conditions (i.e., with smooth, persistent joints) because the intact model was calibrated to reproduce the micromechanical failure mechanisms of the real rock. As such, we expect that joints of other geometries (i.e., density, orientation) and mechanical properties (i.e., persistence, roughness, infilling) could also be added to the BBM and the model would still approximate the behavior of a real specimen with such a joint network.

The intact BBM developed in this study is relatively unique, as it is uncommon for models of intact rock to be calibrated to a large range of mechanical attributes (elastic moduli, CI, CD, peak strength, dilatancy, and brittleness) over a wide range of confining stresses [13,25,26,27,28,29]. However, this rigorous calibration is required to ensure that the micromechanical failure mechanisms of the model approximate those of the real rock [25,26,27,28,29,44,50]. Many of the previous SRMs developed after the jointed Blanco Mera granite specimens (see Table 1) were unable to capture some of the failure mechanisms seen in the jointed Blanco Mera granite laboratory specimens [17,18,19,31,33], in contrast to the more extensively calibrated models developed in this study. For example, in [18,31], the models were not calibrated to match CI and CD thresholds. The results show that, in fact, CD was nearly coincident with peak strength, and we interpret this to have led to a greater confinement dependency of strength than was exhibited by the actual specimens; because of this, peak strength was overestimated under higher confinement levels (6 MPa and above).

Another example is the discrete model developed by [33], which failed predominantly through displacement along the pre-existing joints, rather than through intact material damage, as was observed in the jointed specimens of Blano Mera granite [24]. The strength of the intact model was calibrated to be too high compared to the strength of the pre-existing joints, which resulted in incorrect failure mechanisms of the jointed models.

As noted in Section 3.5, it is not possible to verify the predictive capability of these previously developed models of jointed Blanco Mera granite because their intact and jointed models were calibrated simultaneously. Validation is only possible when the models can be calibrated to an intact data set and then validated using a second data set corresponding to a different condition (i.e., the jointed specimen data). Looking beyond studies of Blanco Mera granite, it is common practice in rock mechanics research to calibrate numerical models to a complete laboratory data set without a separate validation [14,36,79,80,81,82]. Such an approach does not provide any evaluation of the model using data that were not considered as part of the calibration.

There are cases (particularly field-scale scenarios) where a second data set is not always available to verify a model’s predictive capability. In such cases, when utilizing an SRM approach, it is recommended that the model for intact rock be calibrated to a comprehensive set of mechanical attributes, which we interpret to be critical to ensuring the predictive capabilities demonstrated in this study.

Note that this study has only considered one relatively brittle rock with negligible porosity (Blanco Mera granite). The findings of this study will not necessarily apply to more ductile rocks or rocks with non-negligible porosity. Additionally, the numerical models created in this study are two-dimensional, which neglects out-of-plane stress and strains. Further advances in computational capabilities and parallelization of modeling software will make it increasingly feasible to conduct similar studies in three dimensions in the future.

## 8. Conclusions

The idea that a mechanistically accurate numerical model will behave realistically in other scenarios proved to be true for the BBMs created in this study. The addition of pre-existing smooth joints to the intact BBM (with no further calibration) resulted in model behavior that predicted the behavior of the jointed laboratory specimens after which they were modeled without further calibration. Although the BBMs were not able to predict exact values of material attributes, with the exception of a small (i.e., 27% or less) underprediction of peak strength and CD in the case of the higher confinement level values for the 1 + 2 jointing case, the predicted mechanical attribute values were typically within 10% of the model trend derived from the laboratory data.

Two types of BBMs were used in this study: one with blocks modeled via an elastic constitutive model and one with blocks modeled via an inelastic constitutive model. Both BBMs were able to reasonably predict pre-peak attributes and peak strength of the jointed models. However, the pre-peak hardening and post-peak stress–strain behavior were only able to be replicated using the inelastic block BBM. Although the dominant failure mechanism of rock is the formation of fractures along grain boundaries, as significant damage accumulates in rock (particularly after peak strength) intragranular damage becomes increasingly common. This intragranular damage is represented in BBMs by block yield, which is unique to inelastic blocks.

Although the inelastic block BBM was better able to predict the pre-peak hardening and post-peak mechanical behavior of jointed Blanco Mera granite, the elastic and inelastic block BBMs were found to equally predict pre-peak attributes and peak strength of the jointed specimens. Elastic block BBMs are computationally less expensive than inelastic block BBMs, so this is the preferred model type for prediction purposes in cases where accurately simulating post-peak behavior is not critical.

Overall, this study has verified the concept of SRM, where the BBMs were able to replicate the behavior of the rock under new conditions (i.e., with smooth, persistent joints). This finding suggests that joints of other geometries and mechanical properties can also be added to the BBM and the model will approximately predict the mechanical behavior of such a specimen. Since creating rockmass analog laboratory specimens with heavily dense, impersistent, rough, and infilled joints is difficult (if not impossible), the use of numerical models to simulate varying rockmass conditions is a powerful tool for future research into rockmass mechanical behavior.

## Figures and Tables

**Table 2:** The final BBM’s average grain diameters (i.e., equivalent spherical diameter computed based on the area of each block) and proportions for each mineral group compared to the real rock. Table from: [37].

Mineral Group	Rock Proportion	BBM Proportion	Rock Average Grain Diameter (mm)	BBM Average Block Diameter (mm)
Plagioclase	37%	37.03%	3.5	3.50
Quartz	20%	20.18%	3.5	3.47
Alkali Feldspar	27%	27.12%	3.0	2.65
Mica	16%	15.67%	1.9	1.91

**Table 4:** Mineral group densities and elastic parameters assigned to blocks within the BBM. Table from: [37].

Mineral Group	Property	Value	Unit
Plagioclase	Density	2630	kg/m^3^
	Young’s Modulus (E)	55	GPa
	Poisson’s Ratio (ν)	0.18	-
	Bulk Modulus (K)	28.65	GPa
	Shear Modulus (G)	23.31	GPa
Quartz	Density	2650	kg/m^3^
	Young’s Modulus (E)	66	GPa
	Poisson’s Ratio (ν)	0.06	-
	Bulk Modulus (K)	25.00	GPa
	Shear Modulus (G)	31.13	GPa
Alkali Feldspar	Density	2650	kg/m^3^
	Young’s Modulus (E)	42	GPa
	Poisson’s Ratio (ν)	0.15	-
	Bulk Modulus (K)	20.00	GPa
	Shear Modulus (G)	18.26	GPa
Mica	Density	3050	kg/m^3^
	Young’s Modulus (E)	20	GPa
	Poisson’s Ratio (ν)	0.18	-
	Bulk Modulus (K)	10.42	GPa
	Shear Modulus (G)	8.48	GPa

**Table 5:** Contact parameters of the calibrated elastic block BBM. Parameter abbreviations are: jkn = joint normal stiffness, jks = joint shear stiffness, c = cohesion, ϕ = friction angle, jt = tensile strength, c_res_ = residual cohesion, jt_res_ = residual tensile strength, Ψ = dilation angle. Table from: [37].

Property	jkn (GPa/m)	jks (GPa/m)	c (MPa)	Φ (⁰)	jt (MPa)	c_res_ (MPa)	Φ_res_ (⁰)	jt_res_ (MPa)	Ψ (⁰)
Mica-Mica	1.7 × 10^6^	1.1 × 10^6^	38.3	60.0	23.6	0.0	10.0	0.0	10.0
Quartz-Quartz	3.6 × 10^6^	2.4 × 10^6^	62.2	65.0	32.6	0.0	10.0	0.0	10.0
Plagioclase-Plagioclase	3.2 × 10^6^	2.1 × 10^6^	53.6	64.5	34.5	0.0	10.0	0.0	10.0
Feldspar-Feldspar	3.0 × 10^6^	1.9 × 10^6^	52.6	64.5	32.6	0.0	10.0	0.0	10.0
Quartz-Plagioclase	3.0 × 10^6^	1.9 × 10^6^	43.0	62.0	19.6	0.0	10.0	0.0	10.0
Quartz-Mica	3.0 × 10^6^	1.9 × 10^6^	38.3	55.0	35.0	0.0	10.0	0.0	10.0
Plagioclase-Mica	3.0 × 10^6^	1.9 × 10^6^	25.8	55.0	20.9	0.0	10.0	0.0	10.0
Plagioclase-Feldspar	2.7 × 10^6^	1.8 × 10^6^	51.7	62.0	29.8	0.0	10.0	0.0	10.0
Feldspar-Mica	3.0 × 10^6^	1.9 × 10^6^	28.7	55.0	10.6	0.0	10.0	0.0	10.0
Feldspar-Quartz	3.5 × 10^6^	2.3 × 10^6^	36.4	62.0	26.3	0.0	10.0	0.0	10.0

**Table 6:** Inelastic input parameters of the inelastic block BBM that match laboratory data from [24,54]. eps = critical plastic shear strain parameter, which dictates the shear strain at which zones within the blocks decay from peak values to residual.

Inelastic Input Parameter	Value	Units
Peak Cohesion	100	MPa
Peak Friction Angle	57	Degrees
Peak Tensile Strength	40	MPa
Peak Dilation Angle	10	Degrees
Residual Cohesion	50	MPa
Residual Friction Angle	40	Degrees
Residual Tensile Strength	0.5	MPa
Residual Dilation Angle	5.0	Degrees
eps	0.2	-

**Table 7:** Contact parameters of the inelastic block BBM. Parameter abbreviations are: jkn = joint normal stiffness, jks = joint shear stiffness, c = cohesion, ϕ = friction angle, jt = tensile strength, c_res_ = residual cohesion, jt_res_ = residual tensile strength, Ψ = dilation angle.

Property	jkn (GPa/m)	jks (GPa/m)	c (MPa)	Φ (⁰)	jt (MPa)	c_res_ (MPa)	Φ_res_ (⁰)	jt_res_ (MPa)	Ψ (⁰)
Mica-Mica	1.7 × 10^6^	1.1 × 10^6^	35.0	60.0	23.6	0.0	3.0	0.0	7.5
Quartz-Quartz	3.6 × 10^6^	2.4 × 10^6^	33.1	65.0	32.6	0.0	3.0	0.0	7.5
Plagioclase-Plagioclase	3.2 × 10^6^	2.1 × 10^6^	34.9	64.5	34.5	0.0	3.0	0.0	7.5
Feldspar-Feldspar	3.0 × 10^6^	1.9 × 10^6^	38.7	64.5	32.6	0.0	3.0	0.0	7.5
Quartz-Plagioclase	3.0 × 10^6^	1.9 × 10^6^	37.4	62.0	19.6	0.0	3.0	0.0	7.5
Quartz-Mica	3.0 × 10^6^	1.9 × 10^6^	36.4	55.0	35.0	0.0	3.0	0.0	7.5
Plagioclase-Mica	3.0 × 10^6^	1.9 × 10^6^	25.8	55.0	20.9	0.0	3.0	0.0	7.5
Plagioclase-Feldspar	2.7 × 10^6^	1.8 × 10^6^	41.7	62.0	29.8	0.0	3.0	0.0	7.5
Feldspar-Mica	3.0 × 10^6^	1.9 × 10^6^	28.4	55.0	10.6	0.0	3.0	0.0	7.5
Feldspar-Quartz	3.5 × 10^6^	2.3 × 10^6^	35.5	62.0	26.3	0.0	3.0	0.0	7.5

**Table 8:** Input parameters of the pre-existing joints added to the BBM after the calibration process. jkn = joint normal stiffness, jks = joint shear stiffness.

Input Parameter	Value	Units
jkn	750	GPa/m
jks	75	GPa/m
Cohesion	0	MPa
Friction Angle	30	Degrees
Tensile Strength	0	MPa

## Data Availability

For access to the data used in this study, please email the corresponding author, G. Walton at gwalton@mines.edu.

## Appendix A

**Table A1:** Available data on average grain diameters or ranges of diameters for each mineral present in Blanco Mera granite [63] and their proportion in the rock [55]. Note: chlorite exists as twinning within biotite. Table from: [37].

Mineral	Grain Diameter Data	Proportion Percentage
Quartz	1–6 mm	20%
Plagioclase	<6 mm	35%
Alkaline Feldspar	~3.0 mm	27%
Muscovite	~2.5 mm	7%
Biotite w/Chlorite	~1.5 mm	9%
Sericite	<0.2 mm	1%
Accessory Minerals	<0.1 mm	1%

**Table A2:** Mechanical properties of Blanco Mera granite from [24]. Density and tensile strength (σ_T_) were reported in [55] and the Crack Initiation (CI) and Crack Damage (CD) fit parameters as well as Walton–Diederichs (WD) Dilation Parameters [83] were reported in [54]. Note: this value of Young’s Modulus is an averaged value for rock specimens tested at confinements of 12 MPa, as values for Young’s Modulus at lower confinements were interpreted to be artificially reduced by pre-existing flaws within the specimens, induced during specimen extraction [74,84].

	Property	Average Value
Density	Density (g/cm^3^)	2.6
Elastic Properties	E (GPa)	47
	ν	0.17
Tensile Strength	σ_T_ (MPa)	6.12
Hoek–Brown Strength Parameters	σ_ci_ (MPa)	123.4
	m_i_	41.7
	GSI (peak)	100
	GSI (residual)	41.7
Mohr–Coulomb Strength Parameters	UCS (MPa)	132.9
	φ_peak_ (degrees)	59.5
	c_peak_ (MPa)	18.1
	Unconfined Residual Strength (MPa)	15.8
	φ_residual_ (degrees)	50.0
	c_residual_ (MPa)	2.9
Hoek–Brown Crack Damage (CD) Parameters	σ_ci_ (MPa)	77.1
	m_i_	18.6
	GSI	100
Hoek–Brown Crack Initiation (CI) Parameters	σ_ci_ (MPa)	36.7
	m_i_	19.6
	GSI	100
Walton–Diederichs Dilation Model Parameters	α	0–0.1
	γ_m_	1–4
	β_0_	1.0
	β’	0.1
	γ_1_’	−12.2
	γ_2_’	51.6

**Table A3:** Summary of [76] which investigated the effects of inelastic block input parameters on emergent BBM behavior (an increase or decrease in value per the up or down arrows, respectively). Input parameters not listed did not have notable or clear changes in values.

	Effect on Material Attribute (As a Function of Increasing the Block Input Value)			
Inelastic Block Property	Unconfined Strength	Inelastic Block Property	Unconfined Strength	Inelastic Block Property
Peak Cohesion	↑	↑	-	-
Peak Friction Angle	-	↑	-	-
Peak Tensile Strength	↓	↓	-	-
Critical Plastic Shear Strain	-	↑	-	-
Residual Cohesion	-	-	↓	↓
Residual Tensile Strength	-	-	↑	-

**Table A4:** Hoek–Brown fit parameters for intact Peak Strength, Crack Damage (CD), and Crack Initiation (CI) parameters for fits shown in Figure 7.

LAB			
	Strength	CD	CI
**σ_ci_**	132.2	82.3	38.9
**s**	1	1	1
**m_i_**	36.5	16.1	17.3
**a**	0.5	0.5	0.5
**ELASTIC BBM**			
	**Strength**	**CD**	**CI**
**σ_ci_**	116.4	72.2	45.0
**s**	1	1	1
**m_i_**	46.9	20.8	16.2
**a**	0.5	0.5	0.5
**INELASTIC BBM**			
	**Strength**	**CD**	**CI**
**σ_ci_**	167.3	69.3	40.3
**s**	1	1	1
**m_i_**	24.4	24.1	15.4
**a**	0.5	0.5	0.5

**Table A5:** Hoek–Brown fit parameters for 1 + 2 Peak Strength, Crack Damage (CD), and Crack Initiation (CI) parameters for fits shown in Figure 12.

LAB			
	Strength	CD	CI
**σ_ci_**	132.2	82.3	38.9
**s**	1	1	1
**m_i_**	36.5	16.1	17.3
**a**	0.5	0.5	0.5
**ELASTIC BBM**			
	**Strength**	**CD**	**CI**
**σ_ci_**	116.4	72.2	45.0
**s**	0.1	0.6	0.3
**m_i_**	20.8	6.5	3.9
**a**	0.3	0.8	1.0
**INELASTIC BBM**			
	**Strength**	**CD**	**CI**
**σ_ci_**	167.3	69.3	40.3
**s**	0.0	0.3	0.3
**m_i_**	6.2	12.5	3.5
**a**	0.3	0.4	0.8

**Table A6:** Hoek–Brown fit parameters for 2 + 3 Peak Strength, Crack Damage (CD), and Crack Initiation (CI) parameters for fits shown in Figure 13.

LAB			
	Strength	CD	CI
**σ_ci_**	132.2	82.3	38.9
**s**	0.0	0.2	0.3
**m_i_**	10.9	6.1	2.6
**a**	0.4	0.7	1.0
**ELASTIC BBM**			
	**Strength**	**CD**	**CI**
**σ_ci_**	116.4	72.2	45.0
**s**	0.0	0.0	0.0
**m_i_**	10.9	9.0	2.6
**a**	0.4	0.3	0.4
**INELASTIC BBM**			
	**Strength**	**CD**	**CI**
**σ_ci_**	167.3	69.3	40.3
**s**	0.0	0.0	0.0
**m_i_**	5.5	6.8	2.7
**a**	0.4	0.3	0.4

## References

[1] Hoek E., Brown E.T. (1997). Practical estimates of rock mass strength. Int. J. Rock Mech. Min. Sci..

[2] Natau O.P., Frohlich B.O., Muschler T.O. Recent Developments of the Large-Scale Triaxial Test. Proceedings of the ISRM International Symposium.

[3] Muschler T.O., Natau O.P. Further developments for the determination of the stress-strain behaviour of jointed rock mass by large scale tests. Proceedings of the ISRM International Symposium.

[4] Singh M.M., Huck P.J. Large Scale Triaxial Tests on Rock. Proceedings of the 14th US Symposium on Rock Mechanics.

[5] Vergara M.R., Kudella P., Triantafyllidis T. (2015). Large Scale Tests on Jointed and Bedded Rocks Under Multi-Stage Triaxial Compression and Direct Shear. Rock Mech. Rock Eng..

[6] Duan G., Li J., Zhang J., Assefa E., Sun X. (2019). Mechanical Properties and Failure Modes of Rock Specimens with Specific Joint Geometries in Triaxial Unloading Compressive Test. Adv. Mater. Sci. Eng..

[7] Kulatilake P.H.S.W., He W., Um J., Wang H. (1997). A Physical Model Study of Jointed Rock Mass Strength Under Uniaxial Compressive Loading. Int. J. Rock Mech. Min. Sci..

[8] Prudencio M., Van Sint Jan M. (2007). Strength and failure modes of rock mass models with non-persistent joints. Int. J. Rock Mech. Min. Sci..

[9] Ramamurthy T., Arora V.K. (1994). Strength predictions for jointed rocks in confined and unconfined states. Int. J. Rock Mech. Min. Sci..

[10] Shaunik D., Singh M. (2019). Strength behaviour of a model rock intersected by non-persistent joint. J. Rock Mech. Geotech. Eng..

[11] Arzua J., Alejano L.R., Walton G. (2014). Strength and dilation of jointed granite specimens in servo-controlled triaxial tests. Int. J. Rock Mech. Min. Sci..

[12] Mas Ivars D., Pierce M.E., Darcel C. (2011). The synthetic rock mass approach for jointed rock mass modelling. Int. J. Rock Mech. Min. Sci..

[13] Wang X., Cai M. (2020). A DFN—DEM Multi-scale Modeling Approach for Simulating Tunnel Excavation Response in Jointed Rock Masses. Rock Mech. Rock Eng..

[14] Sainsbury B.A., Pierce M., Mas Ivars D. Analysis of Caving Behaviour Using a Synthetic Rock Mass—Ubiquitous Joint Rock Mass Modelling Technique. Proceedings of the First Southern Hemisphere International Rock Mechanics Symposium.

[15] Elmo D., Moffitt K., Carvalho J. Synthetic rock mass modelling: Experience gained and lessons learned. Proceedings of the 50th US Rock Mechanics/Geomechanics Symposium.

[16] Pierce M.E., Cundall P.A., Potyondy D.O., Mas Ivars D. A synthetic rock mass model for jointed rock. Proceedings of the 1st Canada-US Rock Mechanics/Geomechanics Symposium—Rock Mechanics: Meeting Society’s Challenges and Demands.

[17] Bastola S., Cai M. (2020). Investigation of mechanical properties of jointed granite under compression using lattice-spring-based synthetic rock mass modeling approach. Int. J. Rock Mech. Min. Sci..

[18] Shen J., Shu Z., Cai M., Du S. (2020). A shear strength model for anisotropic blocky rock masses with persistent joints. Int. J. Rock Mech. Min. Sci..

[19] Castro-Filgueira U., Alejano L.R., Ivars D.M. (2020). Particle flow code simulation of intact and fissured granitic rock samples. J. Rock Mech. Geotech. Eng..

[20] Suner M.C., Tulu I.B. (2022). Examining the Effect of Natural Fractures on Stone Mine Pillar Strength Through Synthetic Rock Mass Approach. Min. Met. Explor..

[21] Vallejos J.A., Brzovic A., Lopez C., Bouzeran L., Mas Ivars D. Application of the synthetic rock mass approach to characterize rock mass behavior at the El Teniente Mine, Chile. Proceedings of the 3rd International FLAC/DEM Symposium.

[22] Pierce M.E., Mas Ivars D., Sainsbury D. Use of Synthetic Rock Masses (SRM) to Investigate Jointed Rock Mass Strength and Deformation Behavior. Proceedings of the International Conference on Rock Joints and Jointed Rock Masses.

[23] Esmaieli K., Hadjigeorgiou J., Grenon M. (2010). Estimating geometrical and mechanical REV based on synthetic rock mass models at Brunswick Mine. Int. J. Rock Mech. Min. Sci..

[24] Alejano L.R., Arzua J., Bozorgzadeh N., Harrison J.P. (2017). Triaxial strength and deformability of intact and increasingly jointed granite samples. Int. J. Rock Mech. Min. Sci..

[25] Farahmand K., Diederichs M.S. A calibrated synthetic rock mass (SRM) model for simulating crack growth in granitic rock considering grain scale heterogeneity of polycrystalline rock. Proceedings of the 49th US Rock Mechanics/Geomechanics Symposium.

[26] Li X.F., Li H.B., Zhao J. (2019). The role of transgranular capability in grain-based modelling of crystalline rocks. Comput. Geotech..

[27] Park J.W., Park C., Song J.W., Park E.S., Song J.J. (2017). Polygonal grain-based distinct element modeling for mechanical behavior of brittle rock. Int. J. Numer. Anal. Methods Geomech..

[28] Sinha S., Walton G. (2020). A study on Bonded Block Model (BBM) complexity for simulation of laboratory-scale stress-strain be-havior in granitic rocks. Comput. Geotech..

[29] Wang X., Cai M. (2019). A comprehensive parametric study of grain-based models for rock failure process simulation. Int. J. Rock Mech. Min. Sci..

[30] Chen W., Konietzky H. (2014). Simulation of heterogeneity, creep, damage and lifetime for loaded brittle rocks. Tectonophysics.

[31] Huang F., Shen J., Cai M., Xu C. (2019). An Empirical UCS Model for Anisotropic Blocky Rock Masses. Rock Mech. Rock Eng..

[32] Gao G., Meguid M.A. (2022). Microscale Characterization of Fracture Growth in Increasingly Jointed Rock Samples. Rock Mech. Rock Eng..

[33] Gonzalez-Molano N.A., Alvarellos J., Lakshmikantha M.R., Arzua J., Alejano L.R. Numerical and experimental characterization of mechanical behaviour of an artificially jointed rock. Proceedings of the ISRM International Symposium.

[34] Stacey T.R., Wesseloo J. (2022). Design and Prediction in Rock Engineering: The Importance of Mechanisms of Failure, with Focus on High Stress, Brittle Rock Conditions. Rock Mech. Rock Eng..

[35] Zhang X.P., Ji P.Q., Peng J., Wu S.C., Zhang Q. (2020). A grain-based model considering pre-existing cracks for modelling mechanical properties of crystalline rock. Comput. Geotech..

[36] Fan X., Kulatilake P.H., Chen X. (2015). Mechanical behavior of rock-like jointed blocks with multi-non-persistent joints under uniaxial loading: A particle mechanics approach. Eng. Geol..

[37] West I.G., Walton G., Sinha S. Simulating the Behavior of Compressively Loaded Blanco Mera Granite Using Bonded Block Models. Proceedings of the 54th US Rock Mechanics/Geomechanics Symposium.

[38] Turichshev A., Hadjigeorgiou J. (2017). Development of Synthetic Rock Mass Bonded Block Models to Simulate the Behaviour of Intact Veined Rock. Geotech. Geol. Eng..

[39] Garza-Cruz T., Pierce M.E., Board M. (2019). Effect of Shear Stresses on Pillar Stability: A Back Analysis of the Troy Mine Experience to Predict Pillar Performance at Montanore Mine. Rock Mech. Rock Eng..

[40] Garza-Cruz T., Pierce M.E., Kaiser P.K. Use of 3DEC to study spalling and deformation associated with tunnelling at depth. Proceedings of the Seventh International Conference on Deep and High Stress Mining.

[41] Contreras Inga C.E., Walton G., Holley E. (2021). Statistical assessment of the effects of grain-structure representation and micro-properties on the behavior of bonded block models for brittle rock damage prediction. Sustainability.

[42] Lan H., Martin C.D., Hu B. (2010). Effect of heterogeneity of brittle rock on micromechanical extensile behavior during compression loading. J. Geophys. Res..

[43] Li J., Konietzky H., Fruhwirt T. (2017). Voronoi-Based DEM Simulation Approach for Sandstone Considering Grain Structure and Pore Size. Rock Mech. Rock Eng..

[44] Nicksiar M., Martin C.D. (2014). Factors affecting crack initiation in low porosity crystalline rocks. Rock Mech. Rock Eng..

[45] Chen W., Konietzky H., Tan X., Fruhwirt T. (2016). Pre-failure damage analysis for brittle rocks under triaxial compression. Comput. Geotech..

[46] Sinha S., Shirole D., Walton G. (2020). Investigation of the Micromechanical Damage Process in a Granitic Rock Using an Inelastic Bonded Block Model (BBM). J. Geophys. Res. Solid Earth.

[47] Itasca Consulting Group Inc (2014). Universal Distinct Element Code Constitutive Models.

[48] Garza-Cruz T., Pierce M.E. A 3DEC model for heavily veined massive rock masses. Proceedings of the 48th US Rock Mechanics/Geomechanics Symposium.

[49] Ghazvinian E., Diederichs M.S., Quey R. (2014). 3D random Voronoi grain-based models for simulation of brittle rock damage and fabric-guided micro-fracturing. J. Rock Mech. Geotech. Eng..

[50] Wang X., Cai M. (2018). Modeling of brittle rock failure considering inter- and intra-grain contact failures. Comput. Geotech..

[51] Xia Y., Meng Q., Zhang C. (2021). Application of 3D Printing Technology in the Mechanical Testing of Complex Structural Rock Masses. Geofluids.

[52] Yang Z.Y., Chen J.M., Huang T.H. (1998). Effect of joint sets on the strength and deformation of rock mass models. Int. J. Rock Mech. Min. Sci..

[53] Fereshtenejad S., Song J.J. (2021). Applicability of powder-based 3D printing technology in shear behavior analysis of rock mass containing non-persistent joints. J. Struct. Geol..

[54] Walton G., Alejano L.R., Arzua J., Markley T. (2018). Crack Damage Parameters and Dilatancy of Artificially Jointed Granite Samples Under Triaxial Compression. Rock Mech. Rock Eng..

[55] Arzua J., Alejano L.R. (2013). Dilation in granite during servo-controlled triaxial strength tests. Int. J. Rock Mech. Min. Sci..

[56] Lambe T.W. (1973). Predictions in soil engineering. Geotechnique.

[57] Diederichs M.S., Martin C.D. Measurement of spalling parameters from laboratory testing. Proceedings of the European Rock Mechanics EUROCK.

[58] Ghazvinian E., Perras M.A., Diederichs M.S., Labrie D. Formalized approaches to defining damage thresholds in brittle rock: Granite and limestone. Proceedings of the 46th US Rock Mechanics/Geomechanics Symposium.

[59] Martin C.D., Chandler N.A. (1994). The progressive fracture of Lac du Bonnet granite. Int. J. Rock Mech. Min. Sci..

[60] Potyondy D.O. A flat-jointed bonded-particle material for hard rock. Proceedings of the 46th US Rock Mechanics/Geomechanics Symposium.

[61] Potyondy D.O., Cundall P.A. (2004). A bonded-particle model for rock. Int. J. Rock Mech. Min. Sci..

[62] Quey R., Dawson P.R., Barbe F. (2011). Large-scale 3D random polycrystals for the finite element method: Generation, meshing and remeshing. Comput. Methods Appl. Mech. Eng..

[63] University of Vigo, Natural Resources and Environmental Engineering Department, Vigo, Spain. 2011, *Unpublished Petrology Report*.

[64] West I., Walton G. (2023). Quantitative Evaluation of the Effects of Input Parameter Heterogeneity on Model Behavior for Bonded Block Models of Laboratory Rock Specimens. Rock Mech. Rock Eng..

[65] Kazerani T., Zhao J. (2010). Micromechanical parameters in bonded particle method for modelling of brittle material failure. Int. J. Numer. Anal. Methods Geomech..

[66] Stavrou A., Murphy W. (2018). Quantifying the effects of scale and heterogeneity on the confined strength of micro-defected rocks. Int. J. Rock Mech. Min. Sci..

[67] Hoek E., Brown E.T. (1980). Empirical strength criterion for rock masses. J. Geotech. Eng..

[68] Makowski P., Ostrowski Å. (2017). The Methodology for the Young Modulus Derivation for Rocks and Its Value. Procedia Eng..

[69] Gercek H. (2007). Poisson’s ratio values for rocks. Int. J. Rock Mech. Min. Sci..

[70] Clark M.D., Day J.J., Diederichs M.S. Assessing the geomechanical behaviours of skarn-related hydrothermal veins in intact laboratory tests. Proceedings of the 53rd US Rock Mechanics/Geomechanics Symposium.

[71] Ghazvinian E. (2010). Modelling and Testing Strategies for Brittle Fracture Simulation in Crystalline Rock Samples. Ph.D. Thesis.

[72] Zhao X.G., Cai M. (2010). A mobilized dilation angle model for rocks. Int. J. Rock Mech. Min. Sci..

[73] Alejano L.R., Alonso E. (2005). Considerations of the dilatancy angle in rocks and rock masses. Int. J. Rock Mech. Min. Sci..

[74] Walton G., Arzua J., Alejano L.R., Diederichs M.S. (2015). A laboratory-testing-based study on the strength, deformability, and dilatancy of carbonate rocks at low confinement. Rock Mech. Rock Eng..

[75] Walton G., Hedayat A., Kim E., Labrie D. (2017). Post-yield Strength and Dilatancy Evolution Across the Brittle-Ductile Transition in Indiana Limestone. Rock Mech. Rock Eng..

[76] West I., Walton G. Evaluating the Influence of Parameter Inputs on Macroscopic Behavior of Bonded Block Models with Inelastic Blocks. Proceedings of the 56th US Rock Mechanics/Geomechanics Symposium.

[77] Diederichs M.S. (1999). Instability of Hard Rockmasses. Ph.D. Thesis.

[78] Amitrano D. (2006). Rupture by damage accumulation in rocks. Int. J. Fract..

[79] Gao F.Q., Stead D.O. (2014). The application of a modified Voronoi logic to brittle fracture modelling at the laboratory and field scale. Int. J. Rock Mech. Min. Sci..

[80] Chong W.L., Haque A., Gamage R.P., Shahinuzzaman A. (2013). Modelling of intact and jointed mudstone samples under uniaxial and triaxial compression. Arab. J. Geosci..

[81] Yang J.P., Chen W.Z., Yang D.S., Yuan J.Q. (2015). Numerical determination of strength and deformability of fractured rock mass by FEM modeling. Comput. Geotech..

[82] Guo S., Qi S., Zhan Z., Zheng B. (2017). Plastic-strain-dependent strength model to simulate the cracking process of brittle rocks with an existing non-persistent joint. Eng. Geol..

[83] Walton G., Diederichs M.S. (2015). A New Model for the Dilation of Brittle Rocks Based on Laboratory Compression Test Data with Separate Treatment of Dilatancy Mobilization and Decay. Geotech. Geol. Eng..

[84] Vazaios I., Farahmand K., Vlachopoulos N., Diederichs M.S. (2018). Effects of confinement on rock mass modulus: A synthetic rock mass modelling (SRM) study. J. Rock Mech. Geotech. Eng..

